# UBE2J1 inhibits colorectal cancer progression by promoting ubiquitination and degradation of RPS3

**DOI:** 10.1038/s41388-022-02581-7

**Published:** 2022-12-26

**Authors:** Tuo Wang, Chi Jin, Peng Yang, Zhihao Chen, Jiangzhou Ji, Qingyang Sun, Sheng Yang, Yifei Feng, Junwei Tang, Yueming Sun

**Affiliations:** 1 Department of General Surgery, The First Affiliated Hospital of Nanjing Medical University, Nanjing, Jiangsu People’s Republic of China; 2 The First School of Clinical Medicine, Nanjing Medical University, Nanjing, China; 3 The Colorectal Institute of Nanjing Medical University, Nanjing, China; 4 Nanjing Medical University, Nanjing, China

**Keywords:** Metastasis, Ubiquitylation

## Abstract

Ubiquitin-conjugating enzyme E2 J1 (UBE2J1) has been proven to participate in the ubiquitination of multiple substrate proteins. However, the underlying mechanisms of UBE2J1 as a ubiquitin-conjugating enzyme participating in cancer development and progression remain largely unknown. Here, we identified that UBE2J1 is downregulated in colorectal cancer (CRC) tissues and cell lines which are mediated by DNA hypermethylation of its promoter, and decreased UBE2J1 is associated with poor prognosis. Functionally, UBE2J1 serving as a suppressor gene inhibits the proliferation and metastasis of CRC cells. Mechanistically, UBE2J1-TRIM25, forming an E2-E3 complex, physically interacts with and targets RPS3 for ubiquitination and degradation at the K214 residue. The downregulated RPS3 caused by UBE2J1 overexpression restrains NF-κB translocation into the nucleus and therefore inactivates the NF-κB signaling pathway. Our study revealed a novel role of UBE2J1-mediated RPS3 poly-ubiquitination and degradation in disrupting the NF-κB signaling pathway, which may serve as a novel and promising biomarker and therapeutic target for CRC.

## Introduction

Colorectal cancer (CRC) remains one of the most frequent malignancies worldwide ranking third in incidence rate but second in mortality rate [1]. Over 1.9 million newly diagnosed patients and 935,000 deaths caused by CRC were estimated to emerge in 2020 [2]. Surgical excision, radiotherapy as well as chemotherapy, targeted therapy, and immunotherapy are achieved substantial improvement in the therapeutic effect of CRC patients, however, CRC patients with advanced stages still share a bad prognosis [3, 4]. Therefore, the exact molecular mechanisms driving CRC progression and metastasis desperately needed to elucidate, which could provide a prospective biomarker and novel therapeutic target for CRC patients.

The ubiquitin-proteasome system (UPS) is responsible for protein ubiquitination and degradation in eukaryotic cells [5, 6], in which the E1, E2, and E3 enzymes synergistically catalyze the covalent conjugation of ubiquitin to a substrate protein [7, 8]. Moreover, the disorder of UPS is implicated in multiple processes of tumor progression including cell cycle control, angiogenesis, cell proliferation, migration, invasion, and metastasis [9–11]. Belonged to the ubiquitin-conjugating E2 enzyme family, the ubiquitin-conjugating enzyme E2 J1 (UBE2J1) is anchored in the endoplasmic reticulum (ER) membrane and contains a luminal domain, a single transmembrane region, and a cytoplasmic catalytic domain [12]. Up to now, it has been demonstrated that UBE2J1 could induce ubiquitination and degradation of several substrate proteins, which mediate various cellular processes. For example, UBE2J1 was documented to interact with the c-IAP1/TRAF2 (TNF-receptor-associated factor 2) complex to promote TRAF2 ubiquitination [13]. Proteasomal degradation of misfolded MHC I was mediated by UBE2J1 partnered with E3 ligase HRD1 [14]. Similar degradation of misfolded CFTR (cystic fibrosis transmembrane conductance regulator) was catalyzed by the UBE2J1/Derlin-1/RMA1 complex [15]. All the above-mentioned studies show a pivotal role of UBE2J1 in ERAD (ER-associated protein degradation). Furthermore, through facilitating IRF3 (transcription factor IFN regulatory factor 3) ubiquitination, UBE2J1 could promote RNA virus infection via negatively regulating type one IFN expression [16]. However, there is a deficiency of studies on UBE2J1 functioning as a ubiquitin-conjugating enzyme in cancer [17–19].

The nuclear factor κB (NF-κB) is a pleiotropic transcription factor, which serves a pivotal role in the tumorigenesis and progression of human cancers [20, 21]. It has been widely recognized that aberrant activation of NF-κB promotes the proliferation, angiogenesis, chemoresistance, and invasion of tumor cells [22, 23]. Ribosomal protein S3 (RPS3) is a component of the eukaryotic ribosome 40 S subunit, which interacts with elF2 and elF3(eukaryotic initiation factors 2 and 3) to participate in 40 S ribosomal maturation and translation initiation [24]. Moreover, RPS3 possesses multiple extra-ribosomal functions including DNA repair, apoptosis, and radio-resistance et.al [25–27]. The dissociation of the MIF-RPS3 complex induced by radiation activated NF-κB signaling pathway and finally promoted proliferation, inflammation, and metastasis of non-small cell lung cancer (NSCLC) [28]. Most significant is that RPS3 could act as a non-Rel subunit interacting with the p65 subunit of NF-κB via the K homology domain (KH domain) and transcriptionally activate NF-κB induced target gene expression [29]. However, the molecular mechanisms of how RPS3 is regulated in CRC have not been fully elucidated.

In this report, we first revealed that UBE2J1 is downregulated in CRC mediated by its promoter hypermethylation, and diminished UBE2J1 is correlated to poor prognosis. Functionally, UBE2J1 could inhibit the proliferation and metastasis of CRC cells in vitro and in vivo. The UBE2J1-TRIM25 complex induces ubiquitination and degradation of RPS3 at the K214 residue. RPS3 downregulation inhibits NF-κB translocation into the nucleus and therefore inactivates the NF-κB signaling pathway.

## Results

### UBE2J1 is significantly downregulated in CRC and correlated with favorable clinicopathology as well as prognosis

**Fig. 1:**
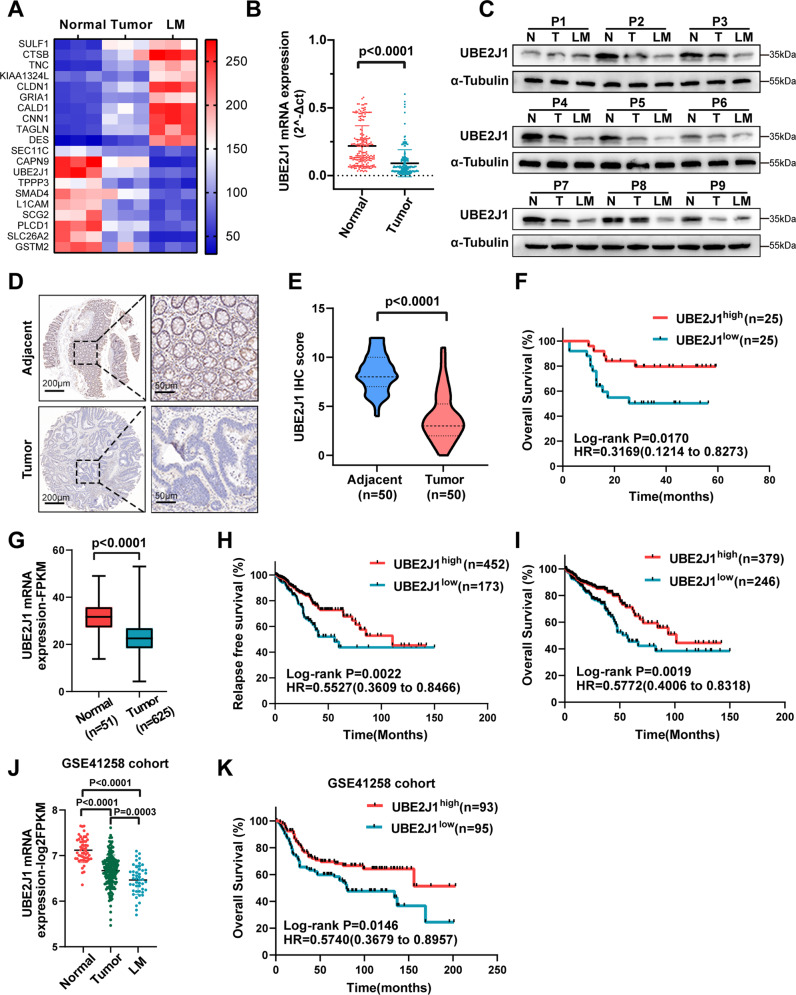
UBE2J1 is downregulated in colorectal cancer and correlated with a favorable prognosis. **A** Heatmap of top 20 upregulated and downregulated proteins among matched liver metastasis (LM), primary tumor, and adjacent normal tissues from 3 CRC patients. **B** The UBE2J1 mRNA level was detected by qRT- PCR in 200 CRC tissues and matched adjacent normal tissues (cohort 1). **C** Protein level of UBE2J1 detected by western blotting in 9 matched liver metastasis, primary tumor, and adjacent normal tissues. **D–F** The protein level of UBE2J1 detected by immunohistochemistry (IHC) in 50 CRC tissues and paired adjacent normal tissues (cohort 2). **D** Representative images of UBE2J1 expression, (**E**) the relative statistical analysis of IHC scores, and (**F**) the Kaplan-Meier survival analysis of patients with UBE2J1^low^ (*n* = 25) or UBE2J1^high^ (*n* = 25) group. **G** Expression of UBE2J1 in the TCGA CRC cohort. **H**, **I** Kaplan-Meier relapse-free survival (RFS) and overall survival (OS) analysis of UBE2J1 expression in patients from TCGA CRC cohort. **J** Expression of UBE2J1 in the GSE41258 CRC cohorts. **K** Kaplan-Meier OS analysis of UBE2J1 expression in patients from GSE41258 CRC cohorts. All data are presented as the means ± SD of three independent experiments and *P* value under 0.05 was considered statistically significant.

To identify the differentially expressed proteins in CRC progression, proteomics sequencing was performed using matched liver metastasis, primary tumor, and adjacent normal tissues from 3 CRC patients (Fig. S1A). Heatmap demonstrated the top 20 upregulated and downregulated proteins and qRT-PCR was used to detect the top 10 differentially expressed proteins in 24 paired tissues (Fig. 1A, S1B). Since UBE2J1 possessed the most dramatic downregulation in 24 paired tissues, it was chosen for subsequent research. To determine the significance of UBE2J1 in CRC, the mRNA and protein levels of UBE2J1 in CRC patients’ samples were detected: 200 samples for qRT-PCR assay (cohort 1), 9 for western blotting assay, and 50 for IHC assay (cohort 2). UBE2J1 expression was remarkably downregulated in CRC samples compared with the paired adjacent normal tissues by using qRT-PCR (Fig. 1B). Metastatic CRC tissues had lower UBE2J1 protein levels than primary CRC tissues and adjacent normal tissues (Fig. 1C). The association between UBE2J1 levels and clinicopathological characteristics of cohort 1 is shown in Table S1. UBE2J1 mRNA expression was negatively correlated to tumor size (*P* = 0.0067), T classification (*P* = 0.0353), TNM stage (*P* = 0.0024), lymph node metastasis (*P* = 0.0125), and distant metastasis (*P* = 0.0112), which indicated that UBE2J1 might play a vital role in modulating proliferation and metastasis of CRC. Furthermore, IHC staining demonstrated that UBE2J1 protein expression was notably lower in CRC tissues than in the adjacent normal counterpart and was mainly located in the cell cytoplasm (Fig. 1D, E). The Kaplan-Meier analysis of cohort 2 revealed that patients with low UBE2J1 expression exhibited unfavorable overall survival (Fig. 1F). Consistent with our above observation, TCGA and GEO database (GSE41258) further confirmed that UBE2J1 is downregulated in CRC tissues and associated with good RFS and OS, which may serve as a potential diagnostic and prognostic biomarker for CRC (Fig. 1G–K).

### UBE2J1 is silenced by promoter CpG methylation in colorectal cancer

**Fig. 2:**
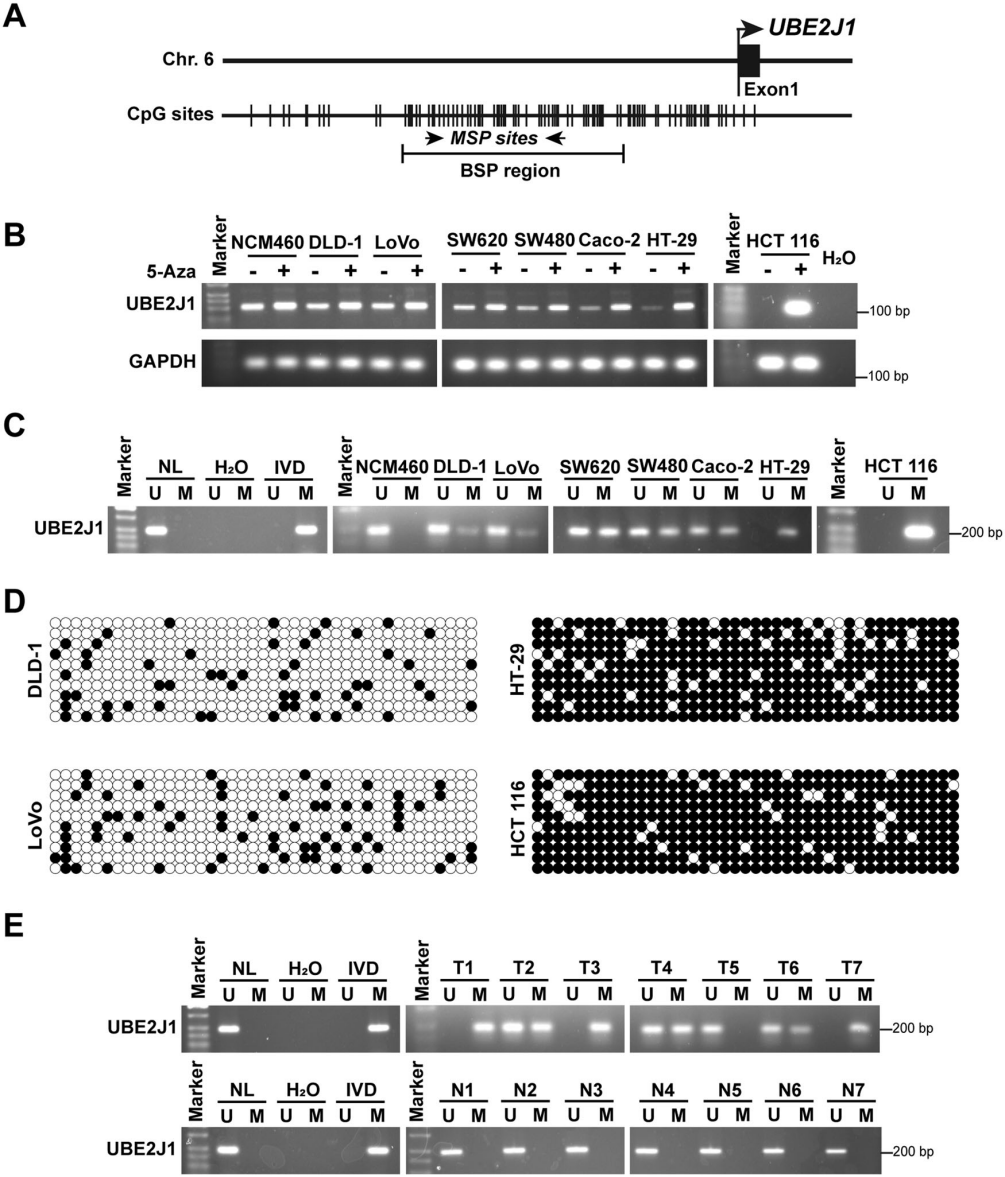
UBE2J1 is silenced by promoter CpG methylation in colorectal cancer. **A** Schematic structure of the UBE2J1 CpG island. CpG sites, MSP sites, and BSP regions are indicated. **B** Expression of UBE2J1 with or without pharmacologic demethylation of 5-Aza was detected by semi-quantitative RT-PCR in a normal colonic epithelial cell and 7 CRC cell lines. *H*_*2*_*O*: double-distilled water, negative control; GAPDH, internal control; 5-Aza: 5-aza-2′-deoxycytidine. **C** MSP results of UBE2J1 in the above cell lines. M: methylated; U: unmethylated; IVD: in vitro methylated DNA, serving as methylation control, NL: normal lymphocytes DNA, serving as unmethylation control. **D** BSP results of UBE2J1 in DLD-1, LoVo, HT-29, and HCT 116 cell lines. Filled circle, methylated CpG site; open circle, unmethylated CpG site. **E** Representative results of MSP for UBE2J1 in primary colorectal cancer samples and matched adjacent tissue samples. T, primary colorectal cancer samples; N, paired adjacent normal tissues. All data are presented as the means ± SD of three independent experiments.

As a tumor suppressor protein, we further explored the mechanism of UBE2J1 downregulation in CRC. Bioinformatic analysis of the UBE2J1 promoter indicated that it contains a typical CpG island (http://www.ebi.ac.uk/Tools/seqstats/emboss_cpgplot/; Fig. 2A), which suggests that the silencing of UBE2J1 may be mediated by promoter CpG methylation. Semi-quantitative RT-PCR showed that UBE2J1 expression was relatively high in NCM460; the levels of UBE2J1 were reduced in SW620, SW480, Caco-2, and HT-29 cells, and lost in HCT 116 cells. Furthermore, the above-mentioned cell lines with downregulation or silencing UBE2J1 were treated with 5-Aza, a DNA methyltransferase inhibitor. A restored expression of UBE2J1 was observed (Fig. 2B). Methylation-specific PCR (MSP) revealed that complete methylation was found in the UBE2J1 promoter region of HT-29 and HCT 116 cells, and partial methylation was observed in the DLD-1, LoVo, SW620, SW480, and Caco-2 cells, and unmethylation was detected in NCM460 (Fig. 2C). Methylation results were reaffirmed by bisulfite sequencing PCR (BSP) in DLD-1, LoVo, HT-29, and HCT 116 cells (Fig. 2D). We further investigated the methylation status of the UBE2J1 promoter by MSP in primary CRC samples and matched adjacent normal tissues. Methylation of UBE2J1 was detected in 71.4% (20/28) of primary colorectal cancers, and no methylation was found in all 28 cases of non-cancerous colorectal tissue samples (Fig. 2E).

### UBE2J1 inhibits CRC cell proliferation and metastasis in vitro and in vivo

**Fig. 3:**
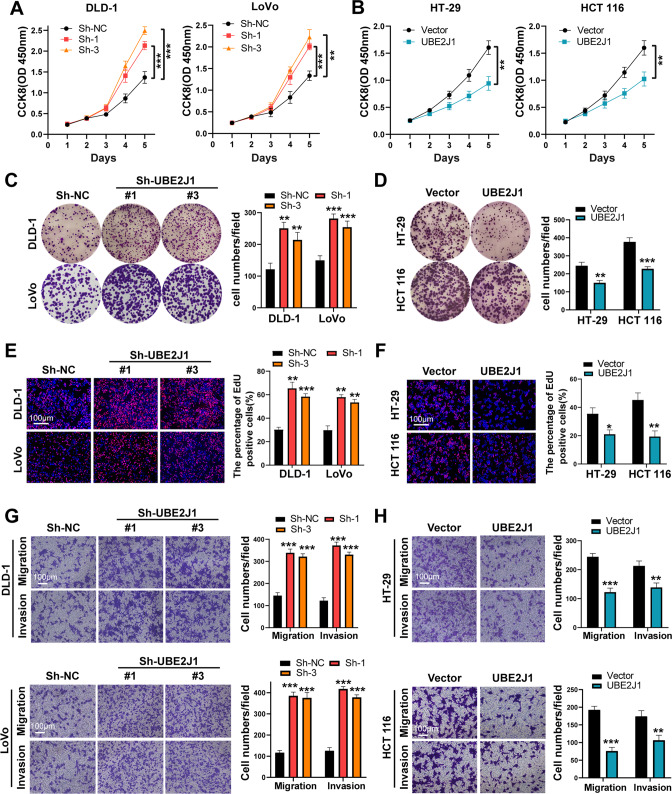
UBE2J1 inhibits CRC cell proliferation and metastasis in vitro. **A**, **B** CCK8 assays were used to detect the viability of UBE2J1 knockdown or overexpression cells. **C**, **D** Colony formation assays were applied to evaluate cell proliferation ability. **E**, **F** EdU staining assays were conducted to assess the proliferation ability of CRC cells. **G**, **H** Transwell assays were performed to evaluate the migration and invasion abilities of CRC cells. All data are presented as the means ± SD of three independent experiments. *P < 0.05, **P < 0.01, ***P < 0.001.

**Fig. 4:**
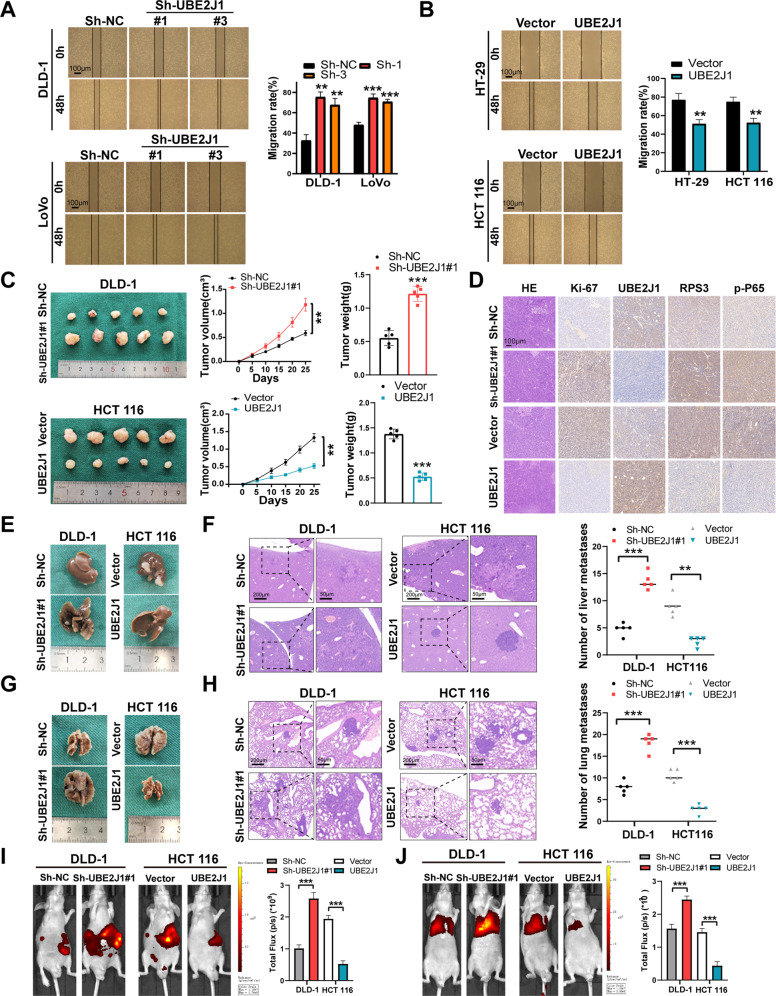
Effects of UBE2J1 on CRC cells proliferation, migration, and invasion in vitro and in vivo. **A**, **B** Wound healing assays were used to assess cell migration ability. **C** Representative photographs of subcutaneous xenograft tumors were obtained from nude mice. Tumor size and average weight were observed. **D** IHC was performed to determine the protein levels of Ki-67, UBE2J1, RPS3, and p-P65 in xenograft tumors. **E**, **G** Representative photographs of liver and lung metastases were obtained from nude mice. **F**, **H** H&E staining of liver and lung metastatic tumors. **I**, **J** Representative images and analysis of bioluminescent intensity in liver and lung metastases are shown. All data are presented as the means ± SD of three independent experiments. **P < 0.01, ***P < 0.001.

To elucidate the biological roles of UBE2J1 in CRC, a series of cell functional assays were performed. qRT-PCR and western blot assays were conducted to detect UBE2J1 expression in seven CRC cell lines and human normal colonic epithelial cells NCM460. The results showed that DLD-1 and LoVo expressed higher levels of UBE2J1, while HT-29 and HCT 116 expressed lower levels of UBE2J1 (Fig. S2A, B). Hence, we knocked down or overexpressed UBE2J1 in the above-mentioned cell lines via lentivirus-mediated infection, respectively. Then, the transfection efficiency of the four cell lines was examined. Sh-UBE2J1#1 and sh-UBE2J1#3 exerted a comparatively better UBE2J1 knockdown efficiency in DLD-1 and LoVo cells and were applied for subsequent experiments (Fig. S2C, D). Cell proliferation ability was evaluated by CCK-8, colony formation, and EdU staining assays. Depletion of UBE2J1 in DLD-1 and LoVo cells yielded expedited growth curves, more and larger colonies formation, and an increased percentage of EdU-positive cells; conversely, UBE2J1 overexpression caused an opposite effect in HT-29 and HCT 116 cells (Fig. 3A–F). Furthermore, transwell and scratch wound healing assays were conducted to measure cell migration and invasion capability. UBE2J1 knockdown prominently promoted the invasion and migration ability of DLD-1 and LoVo cells, whereas UBE2J1 overexpression abated this ability in HT-29 and HCT 116 cells (Figs. 3G, H, and 4A, B). Collectively, these results strongly suggested that UBE2J1 suppresses the proliferation and metastasis of CRC cells in vitro.

To validate whether UBE2J1 affects CRC cell proliferation in vivo, DLD-1 and HCT 116 were exploited to establish UBE2J1 knockdown and overexpression cells via lentivirus transfection. Xenograft tumor models showed that cells with depleted UBE2J1 had a promoting tumor growth, which was reflected in more tumor volume and weight than that in control, while overexpression of UBE2J1 exhibited an opposite effect (Fig. 4C). Moreover, Ki-67 (a proliferation marker), UBE2J1, RPS3, and p-P65 (downstream of UBE2J1) protein levels were measured by IHC staining. Compared to the control group, relatively elevated Ki-67, RPS3, and p-P65 expressions were detected in the UBE2J1 knockdown group. A negative association between UBE2J1 and the above proteins was also observed in the UBE2J1 overexpression group (Fig. 4D). Subsequently, to investigate the effect of UBE2J1 on metastasis in vivo, we constructed a liver and lung metastasis model via the above-mentioned cells. Knockdown of UBE2J1 significantly enhanced the luciferase intensity and increased the number of hepatic as well as pulmonary metastatic nodules, whereas a decreased luciferase intensity and a diminished number of hepatic as well as pulmonary metastatic nodules were measured in UBE2J1 overexpression cells (Fig. 4E–J). These findings illustrated that UBE2J1 suppresses CRC cell proliferation and metastasis in vivo.

### UBE2J1 interacts with RPS3 and negatively regulates its protein level

**Fig. 5:**
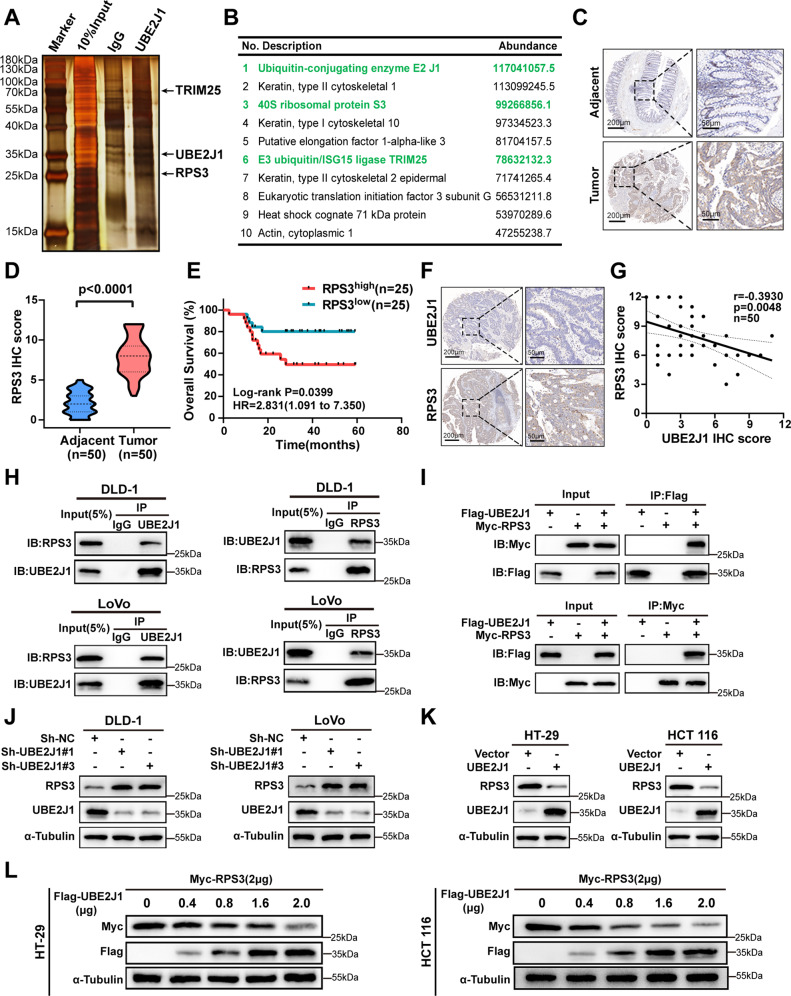
UBE2J1 interacts with RPS3 and negatively regulates its protein level. **A** Silver staining of UBE2J1 immunoprecipitation lysates. Arrows show different bands in immunoprecipitation assays between the UBE2J1 group and IgG group. DLD-1 cells were transfected with UBE2J1 overexpression lentivirus and then divided into input, IgG, and UBE2J1 groups for immunoprecipitation assays. **B** List of the top 10 differentially expressed proteins identified by mass spectrometry. **C**–**E** The protein level of RPS3 was detected by IHC in cohort 2. **C** Representative images of RPS3 expression, (**D**) the relative statistical analysis of IHC scores, and (**E)** the Kaplan-Meier survival analysis of patients with RPS3^low^ (*n* = 25) or RPS3^high^ (*n* = 25) group. **F**, **G** Representative images of IHC staining and correlational analysis between UBE2J1 and RPS3 IHC score in cohort 2. **H** DLD-1 and LoVo cells were treated with MG132 (10 μM) for 8 h and then harvested. Cell lysates were analyzed by co-IP followed by western blotting. **I** HEK293T cells transfected with indicated plasmids for 24 h were treated with MG132 (10 μM) for 8 h. And the exogenous interaction between UBE2J1 and RPS3 was detected by co-IP and western blotting assay. **J**, **K** Four CRC cells with knockdown or ectopic expression of UBE2J1 were collected and then subjected to western blotting. **L** HT-29 and HCT 116 cells were transfected with plasmids encoding Myc-tagged RPS3 and the indicated amounts of Flag-tagged UBE2J1 for 24 h. Cell lysates were analyzed by western blot with indicated antibodies. All data are presented as the means ± SD of three independent experiments and *P* value under 0.05 was considered statistically significant.

To uncover the molecular mechanism underlying the effect of UBE2J1 on CRC suppression, UBE2J1-interacting proteins were identified by mass spectrometry and immunoprecipitation (IP) analyses. Silver staining assay showed that the UBE2J1 immunoprecipitated group was observed with several specific bands of proteins compared to the IgG group (Fig. 5A). The top ten differential proteins were listed and RPS3 had the highest abundance except for cell skeleton protein (Figs. 5B, S2E). A tissue microarray of cohort 2 was analyzed by IHC staining. IHC score showed that RPS3 was more highly expressed in CRC tissues than that in adjacent tissues, which is similar to the expression pattern of RPS3 observed in the TCGA database (Fig. 5C, D, and S2G). Kaplan-Meier plot revealed that patients with high RPS3 expression exhibited reduced overall survival (Fig. 5E). More importantly, the protein levels of RPS3 were negatively correlated with UBE2J1 in CRC tissues (Fig. 5F, G). All these features are contrary to the characteristics of UBE2J1 in CRC patients.

Next, co-immunoprecipitation experiments were employed to verify the physical binding between UBE2J1 and RPS3. Results confirmed that endogenous UBE2J1 is associated with RPS3 and vice versa (Fig. 5H). Furthermore, exogenously expressed UBE2J1 and RPS3 interacting with each other were also observed (Fig. 5I). Since UBE2J1 is a ubiquitin-conjugating enzyme (E2) that mediates ubiquitination and degradation of targeted proteins [12], we surmised that RPS3 might be a ubiquitination substrate of UBE2J1. Knockdown of UBE2J1 with specific shRNAs led to a promotion of endogenous RPS3 protein levels in DLD-1 and LoVo (Fig. 5J). Consistent with this idea, ectopic expression of UBE2J1 significantly down-regulated RPS3 protein levels, and this modulation obeyed a dose-dependent manner in HT-29 and HCT 116 (Fig. 5K, L). whereas, exogenously manipulated UBE2J1 expression did not alter the abundance of RPS3 mRNA levels (Fig. S2H), which suggested that UBE2J1 participates in regulating RPS3 stability through a post-transcriptional mechanism. These results demonstrated that UBE2J1 interacts with RPS3 and regulates its protein levels.

### Poly-ubiquitination and degradation of RPS3 are promoted by UBE2J1 via targeting K214 residue

**Fig. 6:**
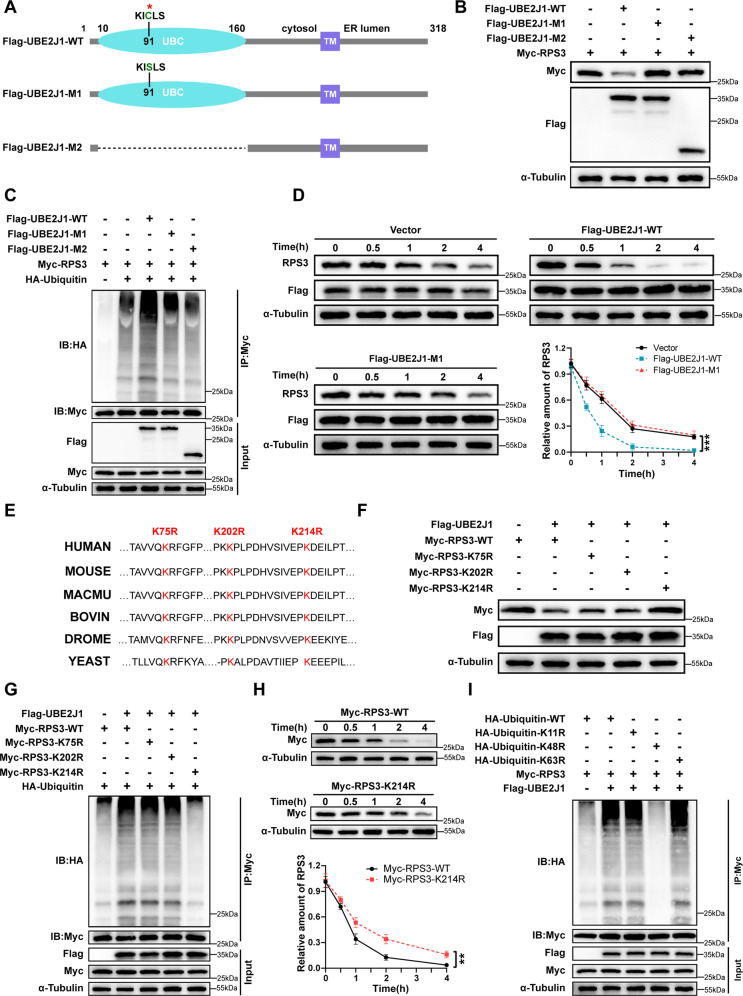
UBE2J1 promotes the poly-ubiquitination and degradation of RPS3 at K214 residue. **A** The schematic diagram of wild-type and mutants UBE2J1. **B** HEK-293T cells were transfected with plasmids encoding Myc-tagged RPS3, along with a plasmid encoding Flag-tagged wild-type UBE2J1 or UBE2J1 mutants (M1, M2). After 24 h, cell lysates were analyzed by western blot with indicated antibodies. **C** HEK-293T cells were transfected with the indicated plasmids. 24 h after transfection, cells were treated with MG132 for 8 h (10 μM). Cell lysates were analyzed by immunoprecipitation with anti-Myc antibodies and western immunoblotting with indicated antibodies. **D** Vector, Flag-tagged wild-type UBE2J1 or UBE2J1 mutant (M1) were transfected into HEK-293T cells for 24 h. Cells were then treated with 100 μg/mL cycloheximide (CHX) and collected for immunoblot analysis at the indicated time points. And quantification of RPS3 band intensity was measured by Image J software. **E** Amino acids sequences alignment of the K75, K202, and K214 in RPS3 from different species. **F** Wild-type and lysine residual mutated Myc-tagged RPS3 plasmids were individually transfected into HEK-293T cells, with or without Flag-tagged UBE2J1. After 24 h, cell lysates were analyzed by western blot with indicated antibodies. **G** HEK-293T cells were transfected with the indicated plasmids for 24 h, followed by treatment with MG132 (10 μM) for 8 h before collection. Cell lysates were subjected to co-IP with an anti-Myc antibody followed by western blotting. **H** HEK-293T cells were transfected with Myc-RPS3-WT or Myc-RPS3-K214R plasmid for 24 h, and then treated with cycloheximide (CHX, 100 μg/mL) for the indicated times before harvesting. Cell lysates were analyzed by immunoblotting with indicated antibodies. **I** HEK-293T cells were transfected with plasmids encoding Flag-tagged UBE2J1 and Myc-tagged RPS3, along with plasmids encoding HA-tagged wild-type ubiquitin or indicated mutant ubiquitin. 24 h after transfection, cells were treated with MG132 for 8 h (10 μM). Cell lysates were analyzed by immunoprecipitation with anti-Myc antibodies and western immunoblotting with indicated antibodies. All data are presented as the means ± SD of three independent experiments. **P < 0.01, ***P < 0.001.

We next sought to explore whether UBE2J1 regulates the stability of RPS3 via ubiquitination. As the cysteine 91 (C91) residue and the ubiquitin-conjugating core (UBC) domain is responsible for the catalytic activity of UBE2J1 [30], we constructed two mutants in which the C91 residue was substituted with a serine (M1, C91S) and the UBC domain was depleted (M2), respectively (Fig. 6A). RPS3 degradation was considerably elevated by ectopic expression of wild-type (WT) UBE2J1, however, the catalytic mutants M1 and M2 failed to do so (Fig. 6B). Meanwhile, UBE2J1 overexpression could prominently induce the poly-ubiquitination of RPS3 compared to the UBE2J1 mutants M1 and M2 (Fig. 6C). In agreement with these findings, WT UBE2J1 overexpression led to a shortened half-life of endogenous RPS3 as compared to the control, whereas UBE2J1 mutant M1 abrogated this trend (Fig. 6D). These data indicated that UBE2J1 serves as a ubiquitin-conjugating enzyme (E2) that induces the ubiquitination and degradation of RPS3.

It has been reported that the ubiquitination of RPS3, as a 40 S subunit of ribosomes, could be regulated by several E3 ubiquitin ligases and deubiquitinates, which participate in Ribosome-Associated Quality Control (RQC) [31–34]. We subsequently intended to discern the potential lysine site of RPS3 that is responsible for UBE2J1-mediated RPS3 ubiquitination. Considering ubiquitin-modified lysine residues are highly conserved across eukaryotes and several specific lysine residues have been documented to be mono-ubiquitination sites of human RPS3 [35], we generated a series of lysine (K) to arginine (R) mutants of RPS3 (K75R, K202R, and K214R) to verify our speculation (Fig. 6E). The ectopic expression of UBE2J1 led to a degradation of RPS3-WT, K75R, and K202R, while the K214R mutant abolished the decrease of RPS3 induced by UBE2J1 overexpression (Fig. 6F). Furthermore, the K214R but not K75R nor K202R mutant dramatically compromised poly-ubiquitination of RPS3 in the presence of UBE2J1 overexpression (Fig. 6G). In line with these observations, the RPS3 K214R mutant possessed an extended half-life than that of WT RPS3 (Fig. 6H). In addition, our data verified that UBE2J1 promotes the K48-linked, but not K11- nor K63-linked poly-ubiquitination of RPS3 (Fig. 6I). Accordingly, these findings provided evidence that UBE2J1 targets RPS3 ubiquitination and degradation via inducing its poly-ubiquitination at K214 residue.

### UBE2J1 suppresses NF-κB translocation into the nucleus and inactivates the NF-κB signaling pathway in CRC by inducing RPS3 degradation

As RPS3 acts as a positive regulator of the NF-κB signaling pathway [29], we were particularly interested in exploring whether UBE2J1 could inactivate the NF-κB signaling pathway via inducing degradation of RPS3. Depletion of UBE2J1 led to a notably increased p-P65 expression in the total cell lysates, promoted accumulation of nuclear P65, as well as enhanced DNA-binding activity of nuclear P65, Whereas RPS3 knockdown abolished these effects caused by the silence of UBE2J1 (Fig. S3A, C). By contrast, ectopic expression of UBE2J1 markedly decreased the amount of p-P65 in the total cell lysates and diminished nuclear P65 levels, and further attenuated the DNA-binding activity of nuclear P65. However, overexpression of RPS3 rescued the above effects which were detected in UBE2J1 overexpressing cells (Fig. S3B, D). Thus, we verified that UBE2J1 suppresses the NF-κB signaling pathway via down-regulating RPS3 expression.

### Ubiquitin E3 ligase TRIM25 cooperates with UBE2J1 to enhance the ubiquitination of RPS3

**Fig. 7:**
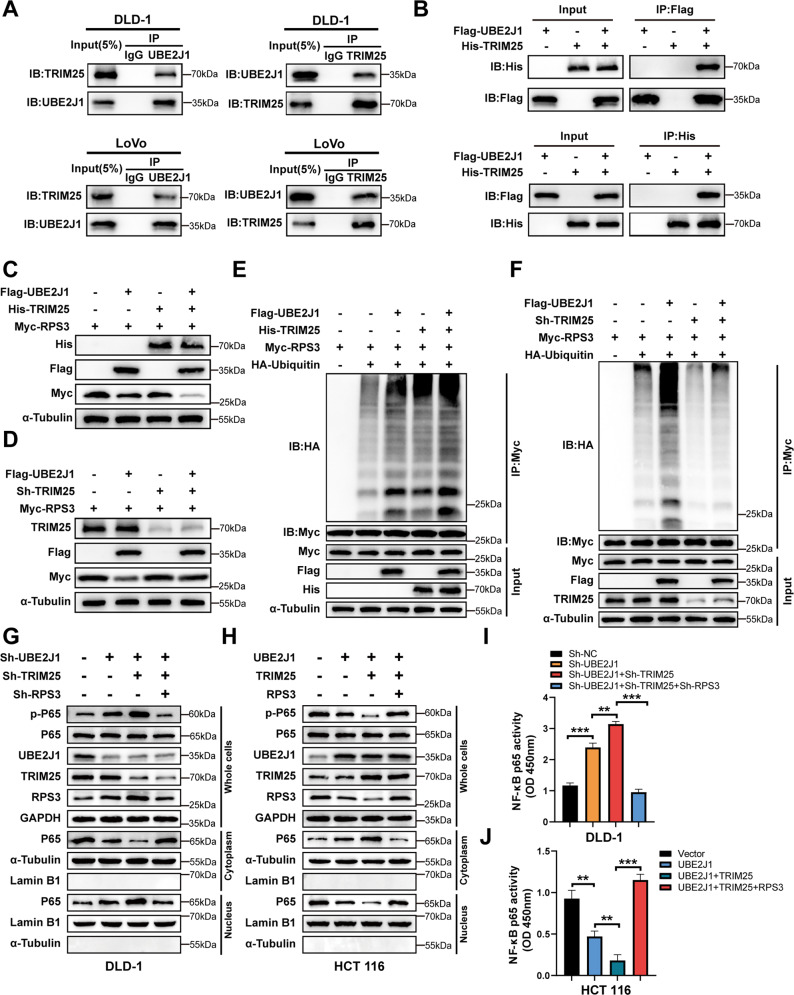
TRIM25 cooperates with UBE2J1 to enhance the poly-ubiquitination and degradation of RPS3 and the TRIM25/UBE2J1-RPS3 axis suppresses NF-κB signaling pathway. **A** The endogenous interaction of UBE2J1 and TRIM25 was tested by co-IP and western blotting assay in DLD-1 and LoVo cells. **B** HEK-293T cells were transfected with indicated plasmids for 24 h. And the exogenous interaction between UBE2J1 and TRIM25 was detected by co-IP and western blotting assay. **C**, **D** Western blotting assay was used to detect the effect of TRIM25 cooperating with UBE2J1 on regulating RPS3 expression. **E**, **F** The effect of TRIM25 cooperating with UBE2J1 on poly-ubiquitination of RPS3. HEK-293T cells expressing the indicated plasmids for 24 h were treated with MG132 for 8 h (10 μM). Cell lysates were immunoprecipitated (IP) with an anti-Myc antibody followed by immunoblotting against indicated antibodies. **G**, **H** Western blot analysis of p‑P65 and P65 protein levels from whole‑cell, nuclear, and cytoplasmic extracts in DLD-1 and HCT 116 cells stably transfected with the indicated lentiviruses. GAPDH, α-Tubulin, and Lamin B1 served as a loading control. **I**, **J** P65 transcription factor DNA binding activity assay in nuclear extracts obtained from DLD-1 and HCT 116 cells stably transfected with the indicated lentiviruses. All data are presented as the means ± SD of three independent experiments. **P < 0.01, ***P < 0.001.

Given that the E2 targeting substrate to ubiquitination and degradation is largely dependent on an E3 ligase [7, 8], we further interrogated the mass spectrometric results to identify an E3 ligase that may partner with UBE2J1. In keeping with our speculation, TRIM25, an E3 ligase with a comparatively high abundance in the UBE2J1-binding proteins pool, was identified (Figs. 5B, S2F). Endogenous and exogenous co-IP experiments demonstrated that UBE2J1 was immunoprecipitated with TRIM25, and vice versa (Fig. 7A, B). Moreover, compared with ectopic expression of UBE2J1 or TRIM25 individually, a more pronouncedly promoting effect on RPS3 degradation and poly-ubiquitination was observed for the overexpression of UBE2J1 and TRIM25 synchronously (Fig. 7C, E). Besides, the knockdown of TRIM25 could diminish RPS3 degradation and poly-ubiquitination caused by the introduction of UBE2J1 (Fig. 7D, F). As a RING-type E3 ligase, TRIM25 belongs to the TRIM proteins family that comprises a RING domain, a coiled-coil (CC) domain, and a PRY/SPRY domain [36]. It has been reported that TRIM25 could serve as a bridge interacting both with E2s and substrates [36–38]. A series of depletion mutants of TRIM25 and RPS3 was constructed to map the critical domains that mediate the interaction between TRIM25 and UBE2J1 or TRIM25 and RPS3 (Fig. S3E, F). As shown in Fig. S3G, H, the coimmunoprecipitation assay demonstrated that the RING domain of TRIM25 and the UBC domain of UBE2J1 mediated the interaction between the two enzymes. Furthermore, the PRY/SPRY domain of TRIM25 and the C-domain of RPS3 were necessary for the binding between TRIM25 and RPS3 (Fig. S3I, J). Next, we asked whether TRIM25 could synergistically promote the effect of UBE2J1 downregulating RPS3 expression and suppressing the NF-κB signaling pathway. Knockdown of TRIM25 and UBE2J1 simultaneously further facilitated the p-P65 nuclear translocation and activated the DNA-binding activity of nuclear P65 compared with the depletion of UBE2J1 only, whereas RPS3 knockdown abrogated these effects (Fig. 7G, I). And the opposite results were observed in the TRIM25 and UBE2J1 overexpression groups (Fig. 7H, J). Thus, we verified that TRIM25 collaborates with UBE2J1, which forms an E2-E3 pair, inducing ubiquitination and degradation of RPS3 and suppressing the NF-κB signaling pathway.

### UBE2J1 impairs the proliferation and metastasis of CRC cells via negatively regulating RPS3

Next, we investigated whether RPS3 is a necessary mediator of the biological functions of UBE2J1 suppressing CRC progression. Stably depleting UBE2J1 cells or control cells were transfected with lentivirus that encodes shRNA-RPS3 or shRNA-control. Meanwhile, UBE2J1, RPS3, or their corresponding vector were also transfected into CRC cells through a lentivirus-mediated system. RPS3 silencing rescued the promoting cell growth caused by UBE2J1 deficiency (Fig. S4A). In contrast, the introduction of RPS3 reversed the decreased cell growth caused by UBE2J1 overexpression (Fig. S4B). Similar results were also observed in the colony formation assay and EdU staining assays (Fig. S4C–F). Transwell assay and Wound healing assay indicated that RPS3 depletion could restore the elevated migration and invasion ability of cells caused by UBE2J1 knockdown, and vice versa (Fig. S5A, B; S6A, B). Collectively, these results showed that UBE2J1 inhibits cell proliferation and metastasis via down-regulating RPS3 in CRC cells.

## Discussion

Accumulating evidence demonstrated that UBE2J1 as an E2 mediates the ubiquitination and degradation of ERAD substrates [13–15]. Nevertheless, rare research showed that UBE2J1 as a ubiquitin-conjugating enzyme is implicated in cancer development and progression. Up to now, it has been documented that prostate cancer patients with high UBE2J1 expression share a relatively poor prognosis [18]. Recently, UBE2J1 is also found to participate in medulloblastoma development, and facilitate endometrial cancer progression [17, 19]. In this study, we first reported that UBE2J1, a ubiquitin-conjugating enzyme, inhibited CRC cell proliferation and metastasis in an RPS3-dependent manner, indicating that UBE2J1 may serve as a tumor suppressor protein that constrains CRC development and progression. Clinically, we found that UBE2J1 expression was remarkably downregulated in CRC patients. Furthermore, decreased UBE2J1 expression was correlated with unfavorable clinicopathology as well as prognosis, which may provide a meritorious biomarker to predict the diagnosis and prognosis of CRC patients.

It has been established that RPS3 expression is relatively higher in colorectal cancer and adenoma, compared to normal colon mucosa [39]. What’s more, depleting RPS3 inhibits proliferation, survival, migration, and invasion and enhances apoptosis of Caco-2 cells [40]. These results are coincident with our study. A relatively increased RPS3 expression of CRC tissues was detected in our cohort and TCGA database. And patients with high RPS3 expression exhibited reduced overall survival. More significantly, the protein level of RPS3 was negatively correlated with UBE2J1 in CRC tissues, suggesting RPS3 may be inversely regulated by UBE2J1.

Several studies have reported that RPS3 serves as a ubiquitination substrate involved in various biological processes. For example, RNF138 induces ubiquitin-dependent degradation of RPS3 and subsequently leads to radioresistance in glioblastoma (GBM) cells [41]. It has been reported that the HSP70/CHIP (carboxy terminus of heat shock protein 70-interacting protein) complex could mediate ubiquitination and subsequent proteasome-dependent degradation of RPS3 [42]. And this phenomenon could be blocked by Heat-shock protein 90 (HSP90), by which HSP90 directly binds to RPS3 [43]. Recently, a study showed that circPLCE1‑411 interacts with the HSP90α/RPS3 complex promoting ubiquitination and degradation of RPS3 to inactivate the NF‑κB pathway in CRC [44]. In the present study, applying proteomics sequencing, we identified a new ubiquitin-conjugating enzyme UBE2J1 as an interacting upstream protein of RPS3, which could facilitate ubiquitin-dependent proteasomal degradation of RPS3 via a post-transcriptional manner. Mass spectrometric results showed that TRIM25, a ubiquitin-ligase, may be a partner molecule that collaborated with UBE2J1. Subsequent experiments demonstrated that TRIM25 cooperating with UBE2J1 downregulated RPS3 protein levels and enhanced poly-ubiquitination of RPS3.

**Fig. 8:**
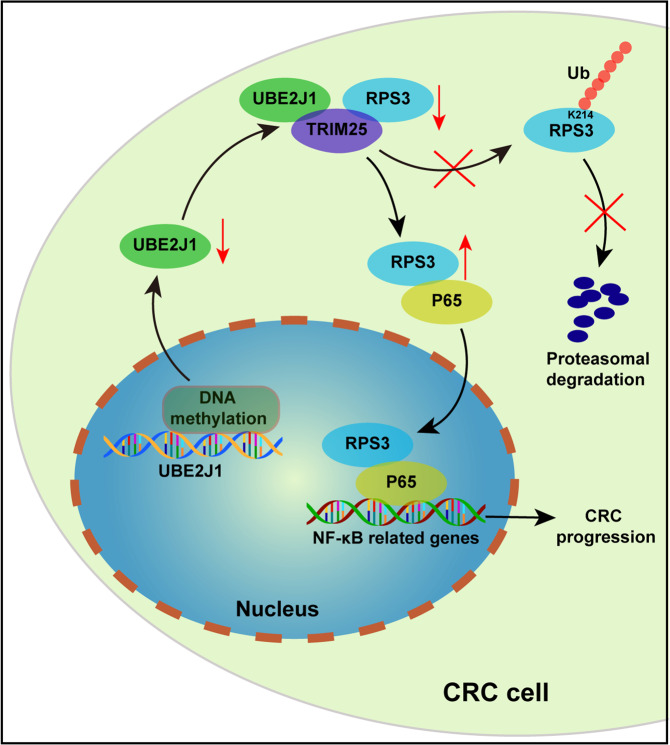
A schematic model for the mechanisms of UBE2J1 in CRC. In summary, our study identified that UBE2J1 as a tumor-suppressor was downregulated in CRC mediated by its promoter hypermethylation. UBE2J1 coupled with TRIM25, targeting RPS3 at K214 residue for poly-ubiquitination and degradation, thereby blocking the activation of the NF-κB signaling pathway.

There is a growing amount of evidence showing that aberrant activated NF-κB signaling plays a vital role in CRC-related processes such as cell proliferation, migration, and metastasis [45–47]. Moreover, accumulation of p65 in CRC tissue is associated with advanced tumor stage and unfavorable overall survival [48, 49]. Given the substantial roles of NF-κB at different stages of CRC progression, it is inspiring that NF-κB inhibitors could come into clinical practice. Several clinical trials have been implemented to assess the therapeutic potential of NF-κB inhibitors in CRC patients [50, 51]. In this study, we experimentally verified that UBE2J1 could suppress p65 nuclear translocation and therefore transcriptionally inactivate the downstream target gene of the NF-κB signaling pathway. And this regulation is dependent on the mediator role of RPS3. Furthermore, our rescue experiments indicated that overexpression of RPS3 could restore decreased accumulation of P65 in the nucleus and attenuated DNA-binding activity of nuclear P65 caused by UBE2J1 deficiency. Function rescue assays also showed that UBE2J1 could inhibit cell proliferation and metastasis via down-regulating RPS3 expression in CRC cells. Our findings identified that the UBE2J1/TRIM25-RPS3-NF-κB axis is a novel pathway mediated CRC proliferation and metastasis, providing a prospective therapeutic target for disrupting this pathway for CRC administration (Fig. 8).

## Methods

### Human specimens and cell culture

All samples were acquired with signed informed consent from the General Surgery Department of the First Affiliated Hospital of Nanjing Medical University. Fresh tissues after surgery were immediately stored at −80 °C conservation. None of the patients received neoadjuvant chemoradiotherapy.

The cell lines DLD-1, LoVo, SW620, SW480, Caco-2, HT-29, HCT 116, NCM460, and HEK-293T were purchased from the Cell Bank of Type Culture Collection of the Chinese Academy of Sciences (Shanghai, China). All cell lines were cultured at 37 °C in a moist atmosphere containing 5% CO_2_.

### Quantitative real-time PCR (qRT-PCR)

TRIzol solution (Invitrogen, USA) was used to extract total RNA according to the manufacturer’s protocols. Total RNA was reversely transcribed to cDNA using HiScript RT Mix (Vazyme, Jiangsu, China). And then, qRT-PCR was conducted using the SYBR Premix Ex Taq Kit (TaKaRa Biotechnology, Dalian, China). The data were analyzed by the StepOne software v2.3. The primers for qRT-PCR are shown in Table S3.

### Cell transfection

The lentivirus containing shRNAs targeting UBE2J1 and RPS3 was synthesized by Obio (Shanghai, China). The full length of UBE2J1 and RPS3 synthesized by Obio were subcloned into the lentivirus vector. The shRNAs targeting TRIM25 and the corresponding negative controls (sh-NC) were synthesized by RiboBio (Guangzhou, China). TRIM25 and ubiquitin overexpression plasmid were obtained from Obio. A series of mutant plasmids of UBE2J1, RPS3, and ubiquitin were synthesized by Obio. Lipofectamine 3000 (Invitrogen) was used for the transfection of shRNAs and plasmid vectors. The transfection efficiency was confirmed by qRT-PCR and immunoblotting. The sequences of shRNAs are listed in Table S3.

### Cell proliferation assays

The Cell Counting Kit-8 (Beyotime, Shanghai, China), colony formation, and 5-ethynyl-2-deoxyuridine (EdU, Beyotime, Shanghai, China) assays were performed as described previously [52]. Proliferation was analyzed using the mean number of cells in three fields for each sample.

### Transwell and scratch wound healing assay

The transwell and scratch wound healing assay was performed as reported previously [53]. Three random fields were selected and measured using microscopy.

### Immunohistochemistry (IHC)

IHC was performed as previously described [52]. Intensity and extent of IHC staining score were used to evaluate protein levels of UBE2J1 and RPS3 (intensity of the staining was assessed on a scale of 0–3: 0, no staining; 1, weak staining; 2, moderate staining; 3, strong staining and the percentage of stained cells was assessed on a scale of 0–4: 0, 0%; 1, 1–24%; 2, 25–49%; 3, 50–74%; 4, 75–100%). The final IHC score is shown by multiplying the positive staining rate and the positive staining area score. Based on the median IHC score, protein expression was sorted into high-expression and low-expression groups. The primary antibodies used are listed in Table S2.

### Protein isolation of nuclear and cytoplasmic fractions

The PARIS™ kit (AM1556; Thermo Fisher Scientific) was used to segregate DLD-1 and HCT 116 into nuclear and cytoplasmic fractions. According to the manufacturer’s protocol, the proteins were isolated from each fraction. The isolated nuclear and cytoplasmic proteins were prepared for the subsequent western blotting assay. GAPDH, α-Tubulin, and Lamin B1 served as internal controls.

### Western blot analysis and antibodies

WB assay was conducted as described previously [54]. The primary antibodies are shown in Table S2.

### Co-immunoprecipitation (Co-IP) assay

IP/Co-IP Kit (#88804, Thermo Fisher Scientific) was used to explore the physical interactions among UBE2J1 with RPS3 and TRIM25. Cell lysates were incubated with a specific primary antibody at 4 °C overnight. The immune complex was incubated with A/G magnetic beads for 1 h and then magnetic beads were washed twice with IP buffer, followed by washing the antigen/antibody complex once with pure water. The products supplemented with 1 × SDS loading buffer were boiled for 10 min. The immunoprecipitated protein was analyzed by Western blot or mass spectrometry (BGI Shenzhen, Guangdong, China). The primary antibody information is listed in Table S2.

### In vivo ubiquitination assay

Cells transfected with indicated plasmids or shRNAs were treated with proteasome inhibitor MG132 (Beyotime, Shanghai, China) for 8 h before collection. Cell lysates were incubated with the anti-Myc antibody for co-immunoprecipitation, and ubiquitinated RPS3 was detected by immunoblotting with an anti-HA antibody.

### 5-Aza-2′-deoxycytidine (5-Aza) treatment

CRC cell lines were split to 30% confluence 12 h before the treatment with 2 μM 5-aza-2’-deoxycytidine (5-Aza, Sigma, MO, USA). The 5-Aza was replenished every day for a total of 4 days. Then, RNA was extracted from the treated cells as described above.

### Methylation-specific PCR (MSP) and Bisulfite sequencing PCR (BSP) analysis

DNeasy Blood & Tissue kit (Qiagen) was used to extract the genomic DNA of CRC cell lines. Then, 2 μg of the collected genomic DNA were incubated with bisulfite DNA Lysis Buffer for 1 h at 37 °C. The samples were denatured, and bisulfite deaminated. Bisulfite-treated DNA was PCR-amplified by primers of MSP, and PCR products were visualized by agarose gel electrophoresis. The bisulfite-treated genomic DNA was PCR-amplified by primers of BSP. PCR products were then purified and cloned into pMD19-T (TaKaRa, Dalian, China). The cloned products were used for DNA sequencing. Primer sequences for MSP and BSP are listed in Table S3.

### Animal models

Five-week-old male BALB/c nude mice were used for the subcutaneous tumor formation and liver, and lung metastasis model. For the xenograft model, 1 × 10^6^ DLD-1 cells stably transfected with sh-UBE2J1 and HCT 116 cells stably expressing UBE2J1 along with corresponding control cells were separately injected into the left and right groins of the mice. The tumor volumes and weights were measured every 5 days. Twenty-five days after the subcutaneous injection, mice were sacrificed and the xenograft tumors were dissected and subjected to H&E and IHC staining. For liver and lung models, 1 × 10^6^ luciferase-labeled above-mentioned cells were injected into the distal tip of the spleen or implanted into the blood through a tail vein. After 4 weeks, the mice received intraperitoneal injections of 150 mg/kg of D-luciferin (Goldbio, USA), and images were acquired 10 min after injection with an IVIS 100 Imaging System (Xenogen, Hopkinton, MA, USA). All mice were sacrificed and the liver, as well as lung tissues, were dissected and observed through H&E staining. All animal experiments were approved by the Committee on the Ethics of Animal Experiments of Nanjing Medical University.

### Statistical analysis

GraphPad Prism 9.0 (La Jolla, CA, USA) and SPSS 13.0 (Chicago, IL, USA) were used for statistical analyses. The student’s *t* test, ANOVA, Chi-square test, and Kaplan-Meier analysis were applied for statistical comparisons in this study. Each experiment was repeated more than three times. Data are shown as means ± standard deviation (SD). *P* < 0.05 was considered statistically significant.

## Supplementary information

Supplementary information

Figure S1

Figure S2

Figure S3

Figure S4

Figure S5

Figure S6

Figure S7

Table S1

Table S2

Table S3

Original western blots

## Data Availability

The data sets used in the current study are available from the corresponding author on reasonable request.
