# Supplementary material for: UBE2J1 inhibits colorectal cancer progression by promoting ubiquitination and degradation of RPS3
Source: Oncogene. 2022 Dec 26;42(9):651–64. doi: 10.1038/s41388-022-02581-7 (PMC9957728; doi:10.1038/s41388-022-02581-7)
Supplement: Supplementary file 1 — Supplementary information [file 41388_2022_2581_MOESM1_ESM.docx]

**Supplementary information**

**Additional file 1: Table S1.** Association of UBE2J1 expression with clinicopathological factors (n=200).

**Additional file 2: Table S2.** Antibodies were used in this study.

**Additional file 3: Table S3.** qRT-PCR, MSP, BSP primers, and shRNAs were used in this study.

**Additional file 4: Fig. S1.** The screening of UBE2J1. **A.** proteomics sequencing results (with fold change >1.2 and p <0.05) were plotted as a volcano plot. **B.** The levels of the top 10 upregulated and downregulated proteins were detected by qRT-PCR in 24 cases of patients with matched liver metastasis, primary tumor, and adjacent normal tissues. All data are presented as the means ± SD of three independent experiments and a *P*-value under 0.05 was considered statistically significant. ^ns^*P* > 0.05.

**Additional file 5: Fig. S2.** The construction of UBE2J1 stably knockdown and overexpression CRC cell lines, and exploring UBE2J1 mediated RPS3 regulation. **A. and B.** Relative expression of UBE2J1 was detected by qRT-PCR and western blotting in CRC cell lines and normal epithelial colon cell NCM460. **C. and D.** qRT-PCR and western blotting were used to measure the knockdown and overexpression efficiency of UBE2J1 in selected CRC cell lines. **E. and F.** The identified RPS3 and TRIM25 were by mass spectrometry analysis. **G.** Expression of RPS3 in the TCGA CRC cohort. **H.** The mRNA expression level of RPS3 in UBE2J1 knockdown and overexpression cells. All data are presented as the means ± SD of three independent experiments and a *P*-value under 0.05 was considered statistically significant. ^ns^*P* > 0.05, ***P* < 0.01, ****P* < 0.001.

**Additional file 6: Fig. S3.** The UBE2J1-RPS3 axis suppresses NF-κB signaling pathway and TRIM25 interacts both with UBE2J1 and RPS3. **A. and B.** Western blot analysis of p‑P65 and P65 protein levels from whole‑cell, nuclear, and cytoplasmic extracts in DLD-1 and HCT 116 cells stably transfected with the indicated lentiviruses. GAPDH, α-Tubulin, and Lamin B1 served as a loading control. **C. and D.** P65 transcription factor DNA binding activity assay in nuclear extracts obtained from DLD-1 and HCT 116 cells stably transfected with the indicated lentiviruses. **E.** Schematic diagram of full-length TRIM25 and truncated mutants. **F.** Schematic of RPS3 and different truncations. RPS3 contains an N-domain (ND) comprising amino acids 1–92, a C-domain (CD) constituting amino acids 93–199, and a C-terminal domain (CTD) (amino acids 200–243). Among this, the N-domain of RPS3 contains a type II KH domain. **G. and H.** Mapping of the TRIM25-UBE2J1 interaction. HEK293T cells were co-transfected with the indicated His-TRIM25 and Flag-UBE2J1 or indicated Flag-UBE2J1 and His-TRIM25. Cell extracts were immunoprecipitation with an anti-His antibody or anti-Flag antibody and then immunoblotted with the indicated antibodies. **I. and J.** Mapping of the TRIM25-RPS3 interaction. HEK293T cells were co-transfected with the indicated His-TRIM25 and Myc-RPS3 or indicated Myc-RPS3 and His-TRIM25. Cell extracts were immunoprecipitation with an anti-His antibody or anti-Myc antibody and then immunoblotted with the indicated antibodies. All data are presented as the means ± SD of three independent experiments. ***P* < 0.01.

**Additional file 7: Fig. S4.** UBE2J1 suppresses the proliferation ability of CRC cells through downregulating RPS3. **A-F.** CCK-8, colony formation, and EdU assays were carried out in CRC cells stably transfected with the indicated lentiviruses. All data are presented as the means ± SD of three independent experiments. ***P* < 0.01, ****P* < 0.001.

**Additional file 8: Fig. S5.** UBE2J1 inhibits the metastasis ability of CRC cells through RPS3. **A. and B.** Transwell assays were performed in CRC cells stably transfected with the indicated lentiviruses. All data are presented as the means ± SD of three independent experiments. **P* < 0.05, ***P* < 0.01, ****P* < 0.001.

**Additional file 9: Fig. S6.** UBE2J1 inhibits the metastasis ability of CRC cells through RPS3. **A. and B.** Wound healing assays were conducted in CRC cells stably transfected with the indicated lentiviruses. All data are presented as the means ± SD of three independent experiments. ***P* < 0.01.

**Additional file 10: Fig. S7. A. and B.** The knockdown and overexpression efficiency of RPS3 and TRIM25 was detected by qRT-PCR and Western blotting. **C. and D.** UBE2J1 and RPS3 IHC results of a tissue microarray containing 50 cases of the primary tumor, and matched adjacent normal tissues. All data are presented as the means ± SD of three independent experiments. ***P* < 0.01, ****P* < 0.001.
