# Supplementary figures and images for: UBE2J1 inhibits colorectal cancer progression by promoting ubiquitination and degradation of RPS3
Source: Oncogene. 2022 Dec 26;42(9):651–64. doi: 10.1038/s41388-022-02581-7 (PMC9957728; doi:10.1038/s41388-022-02581-7)

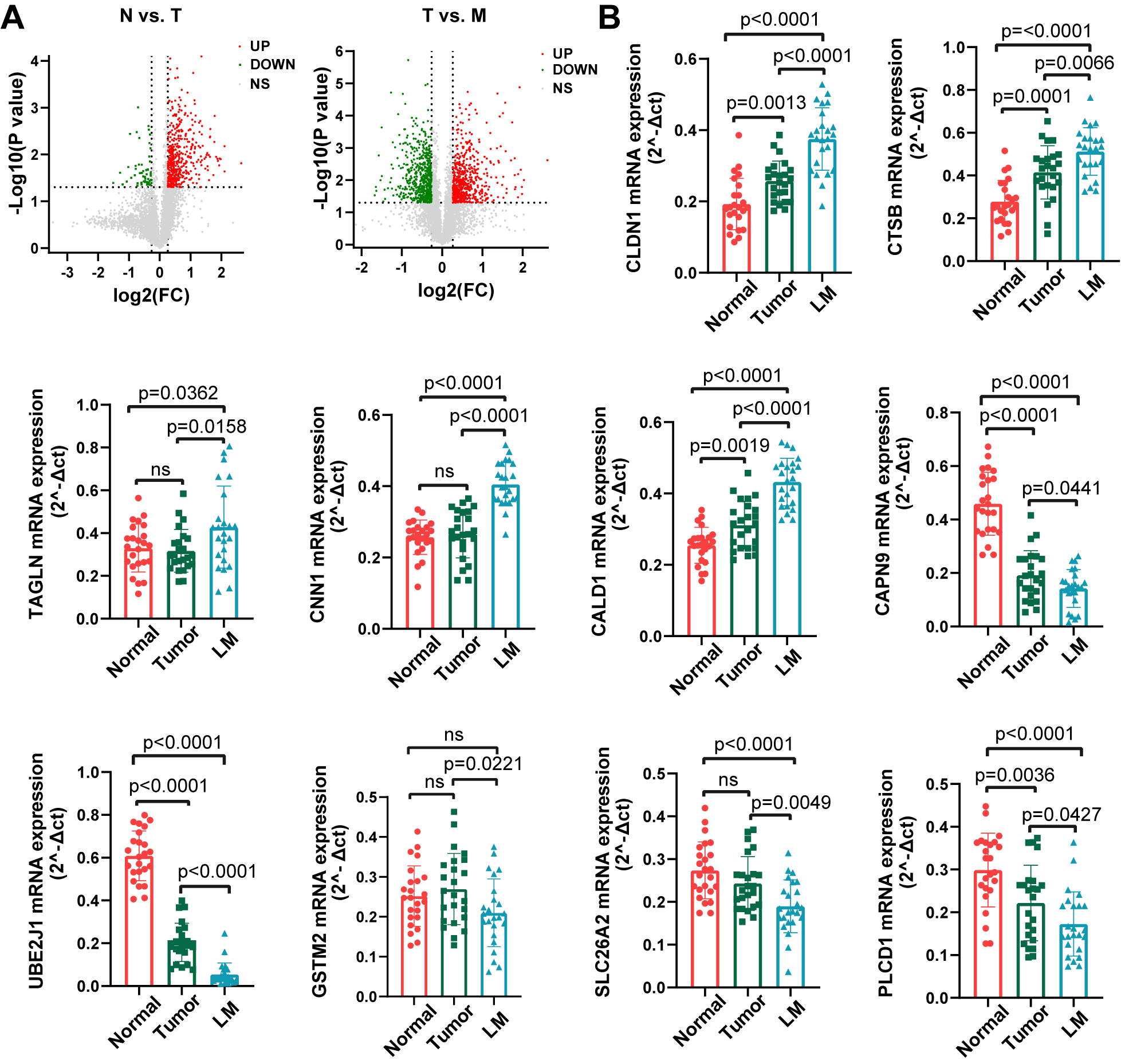

Supplement: Supplementary file 2 — Figure S1 [file 41388_2022_2581_MOESM2_ESM.tif]

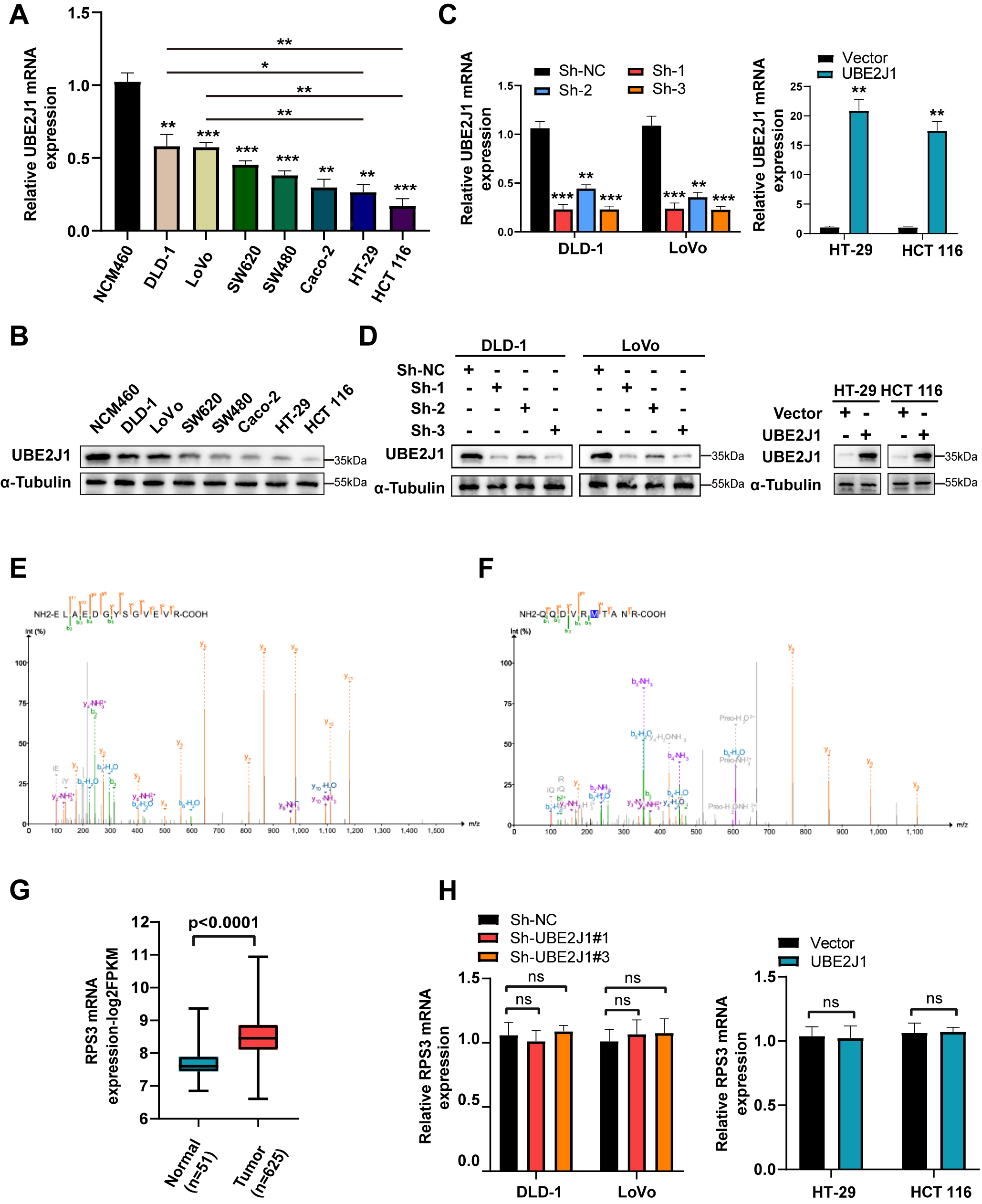

Supplement: Supplementary file 3 — Figure S2 [file 41388_2022_2581_MOESM3_ESM.tif]

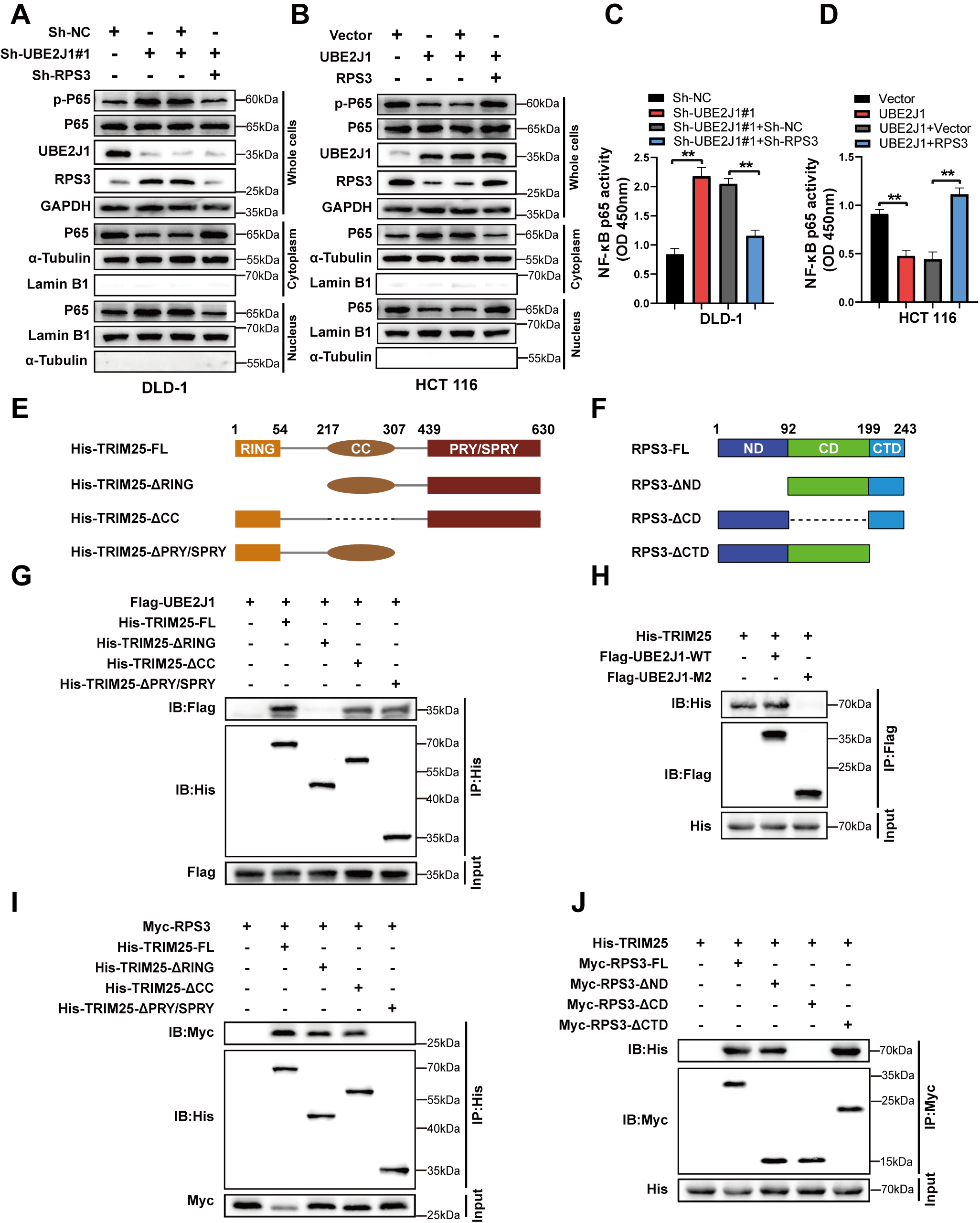

Supplement: Supplementary file 4 — Figure S3 [file 41388_2022_2581_MOESM4_ESM.tif]

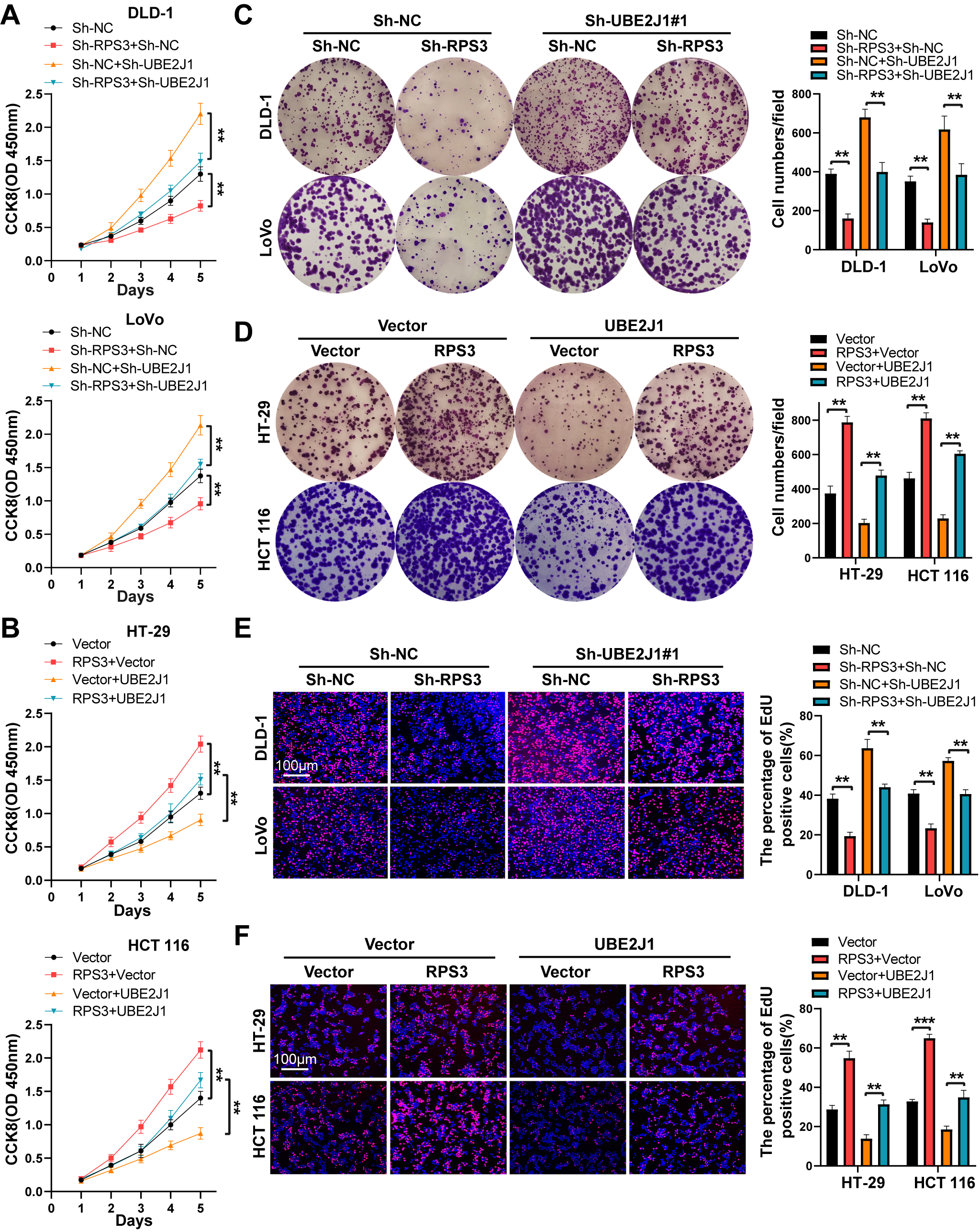

Supplement: Supplementary file 5 — Figure S4 [file 41388_2022_2581_MOESM5_ESM.tif]

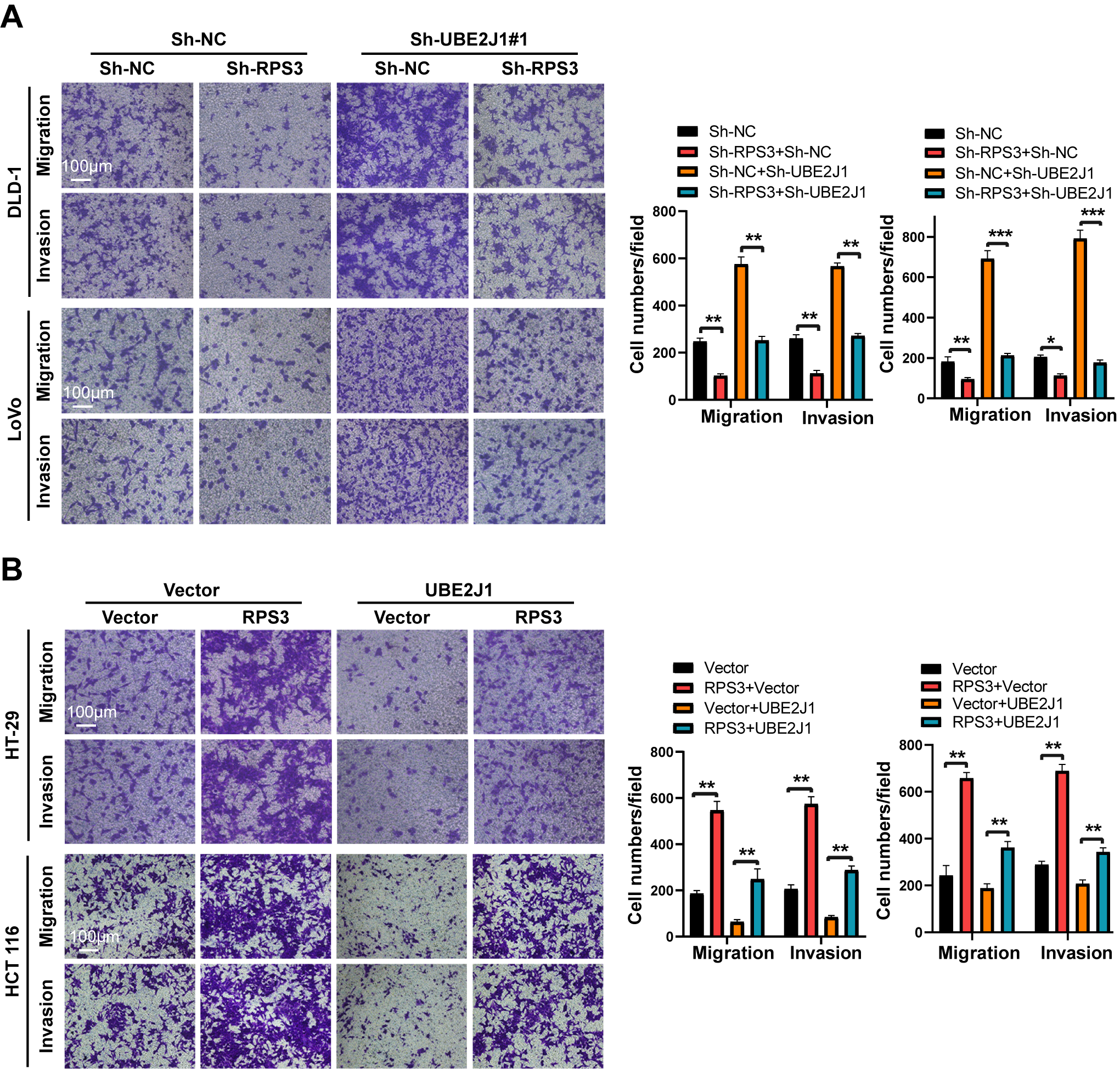

Supplement: Supplementary file 6 — Figure S5 [file 41388_2022_2581_MOESM6_ESM.tif]

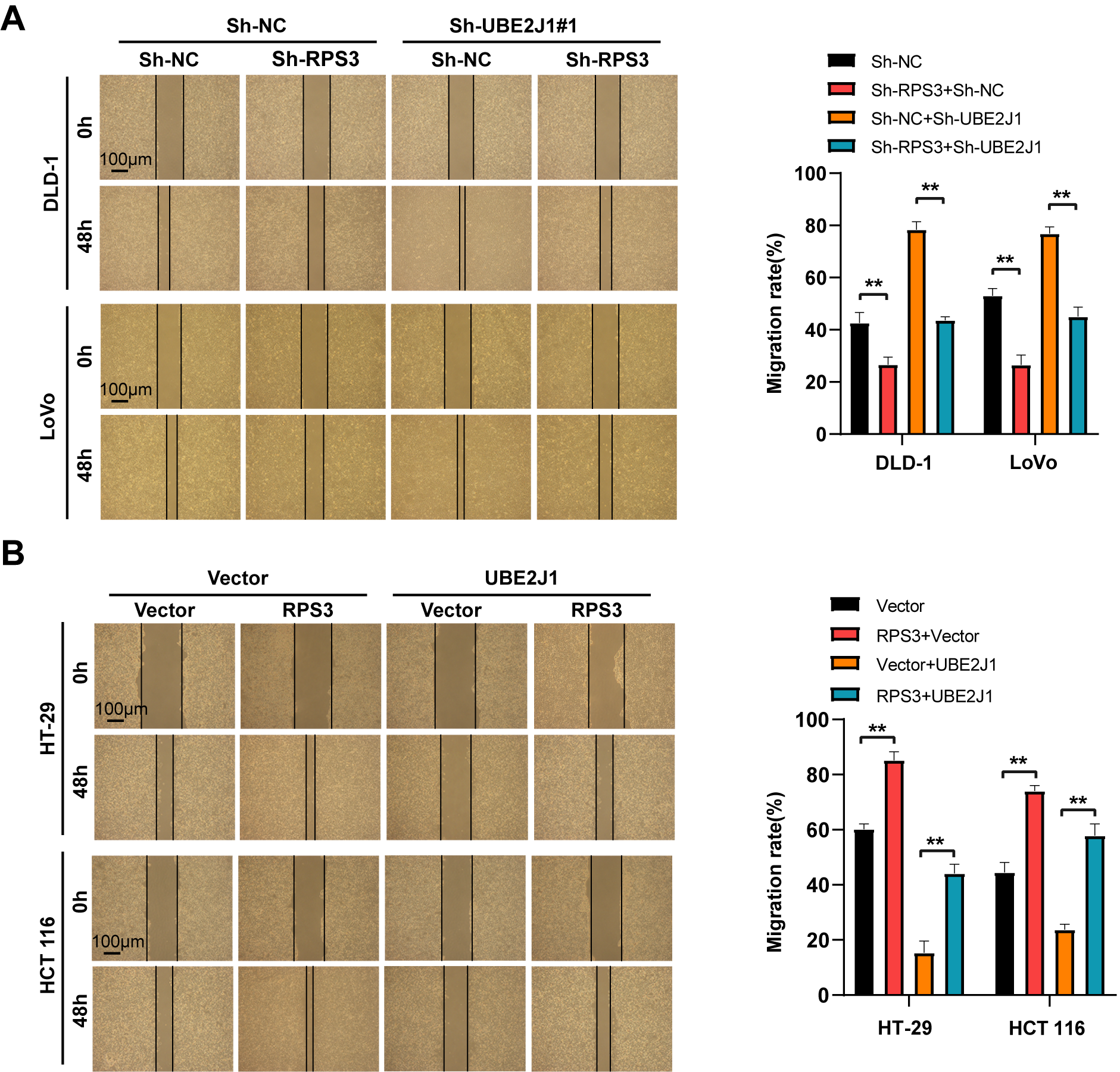

Supplement: Supplementary file 7 — Figure S6 [file 41388_2022_2581_MOESM7_ESM.tif]

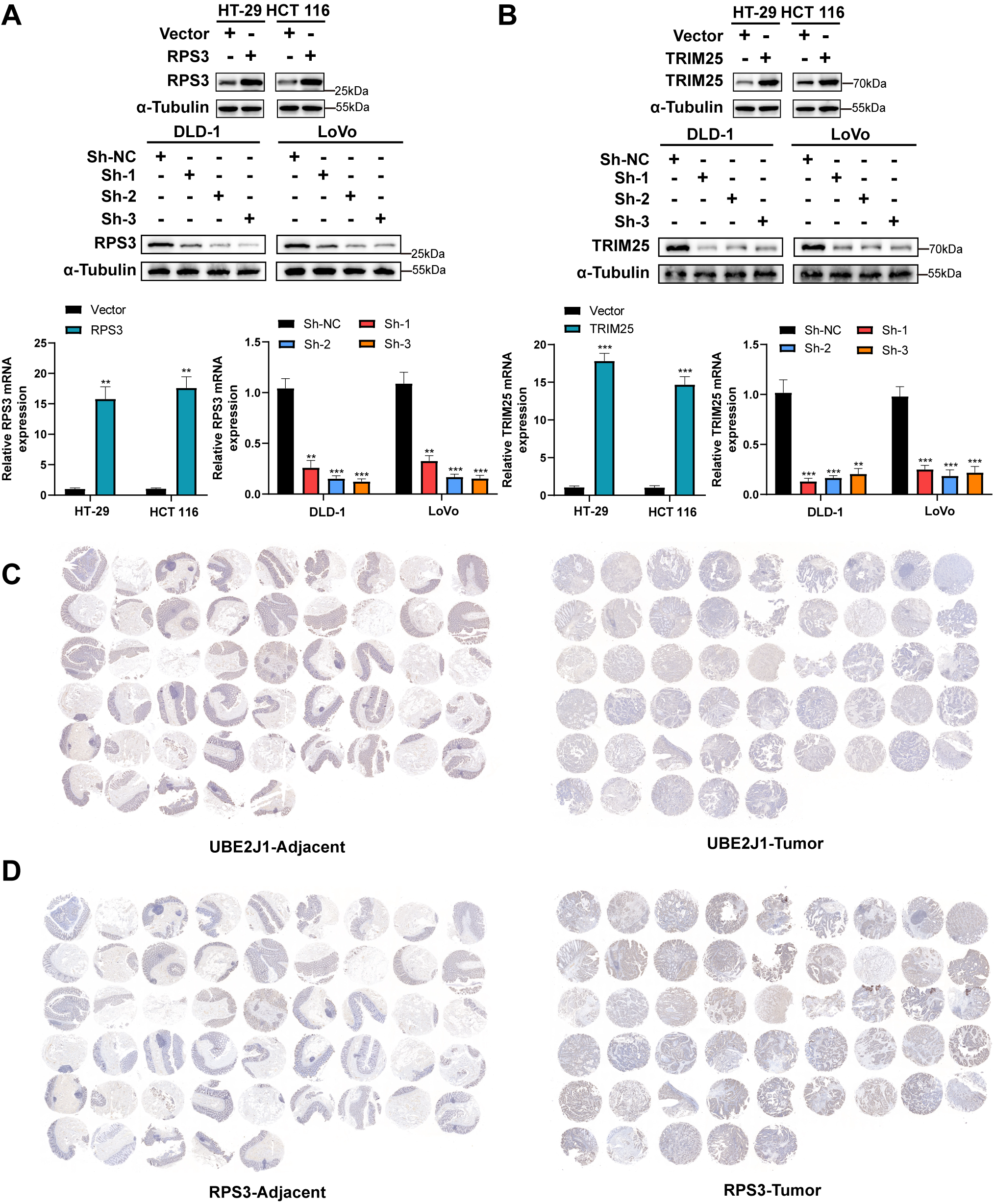

Supplement: Supplementary file 8 — Figure S7 [file 41388_2022_2581_MOESM8_ESM.tif]

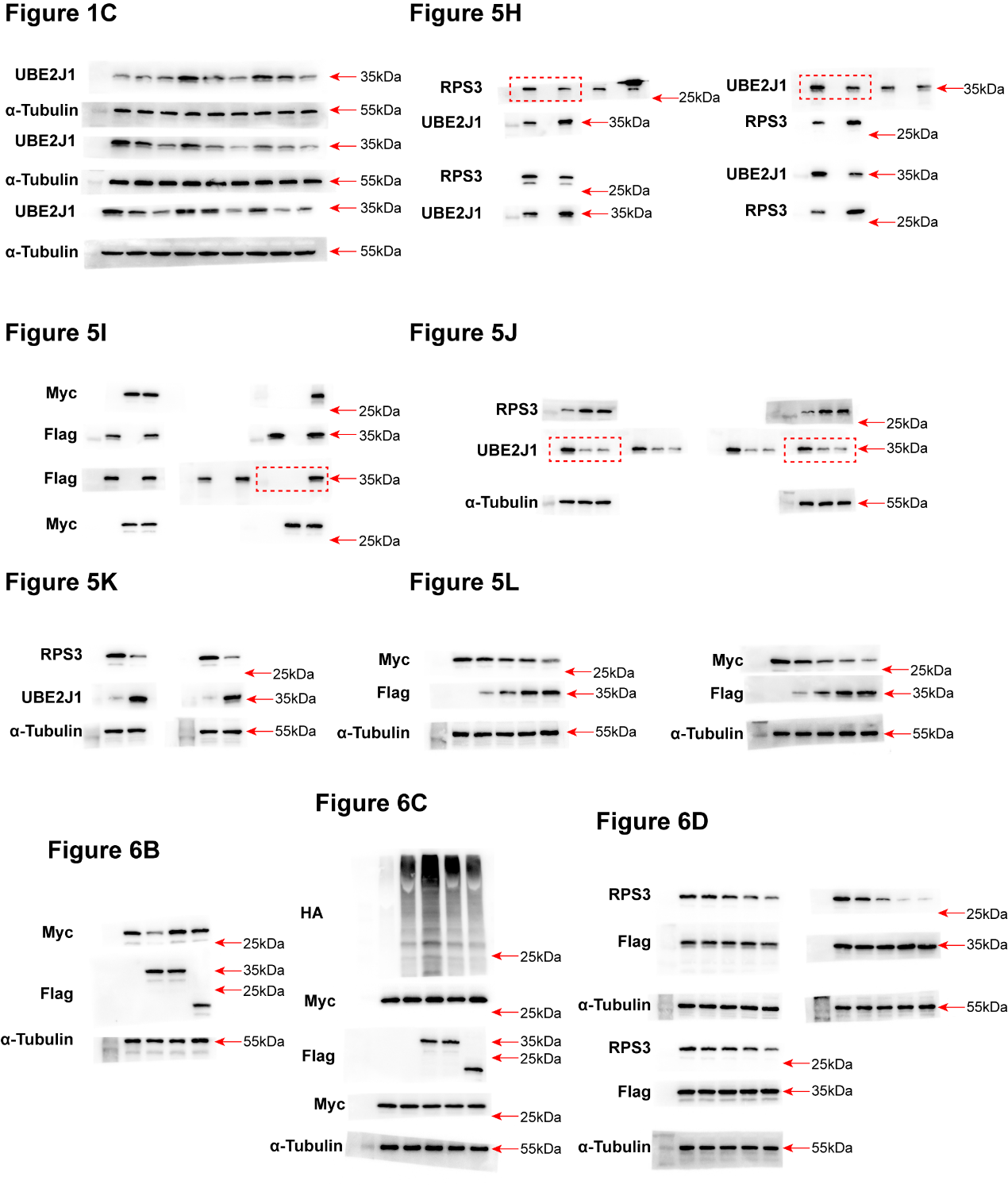


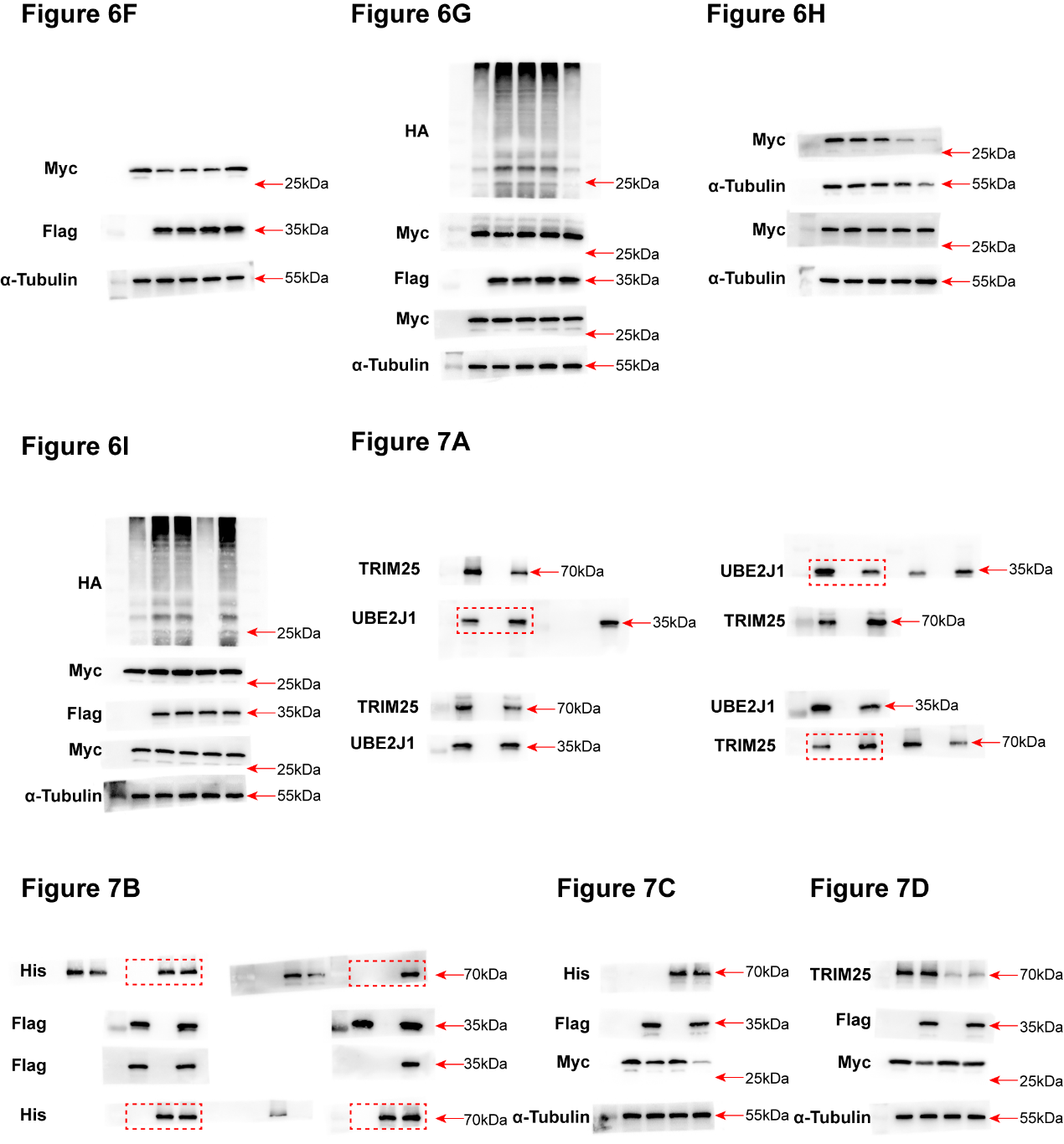


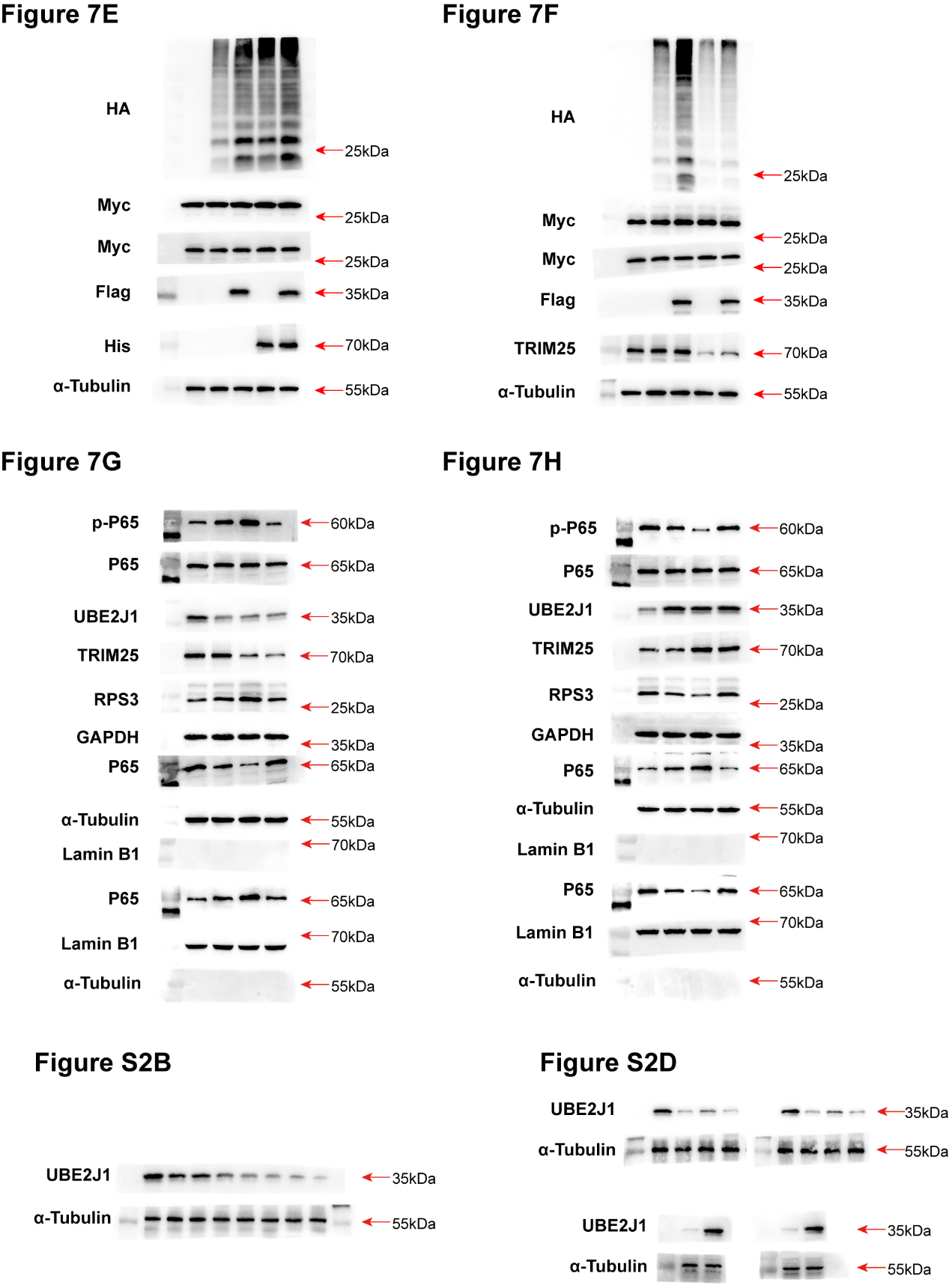


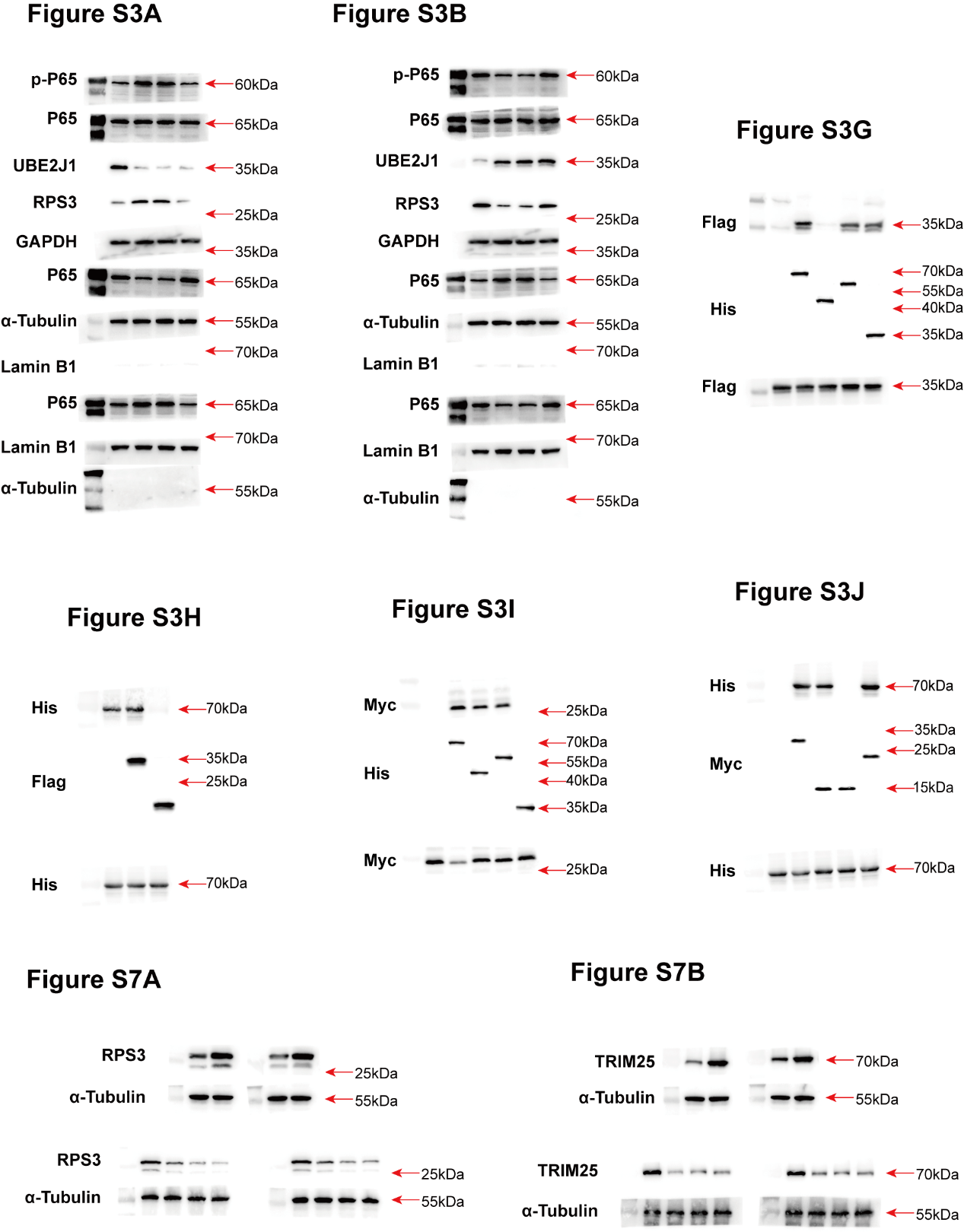

Supplement: Supplementary file 12 — Original western blots [file 41388_2022_2581_MOESM12_ESM.docx]
