# Supplementary material for: UBE2J1 inhibits colorectal cancer progression by promoting ubiquitination and degradation of RPS3
Source: Oncogene. 2022 Dec 26;42(9):651–64. doi: 10.1038/s41388-022-02581-7 (PMC9957728; doi:10.1038/s41388-022-02581-7)
Supplement: Supplementary file 9 — Table S1 [file 41388_2022_2581_MOESM9_ESM.docx]

| **Table S1 Association of UBE2J1 expression with clinicopathological factors(n=200)** | | | | |
| --- | --- | --- | --- | --- |
| **Variable** | **All patients** | **Expression of UBE2J1** | | **P value** |
|  |  | **High** | **Low** |  |
| All Cases | 200 | 100 | 100 |  |
| Age (years) |  |  |  |  |
| <60 | 53 | 29 | 24 | 0.4231 |
| ≥60 | 147 | 71 | 76 |  |
| Gender |  |  |  |  |
| Male | 120 | 59 | 61 | 0.7728 |
| Female | 80 | 41 | 39 |  |
| Tumor size (cm) |  |  |  |  |
| <5 | 87 | 53 | 34 | **0.0067** |
| ≥5 | 113 | 47 | 66 |  |
| T classification |  |  |  |  |
| T1 + T2 | 66 | 40 | 26 | **0.0353** |
| T3 + T4 | 134 | 60 | 74 |  |
| TNM stage (AJCC) |  |  |  |  |
| Stage I+II | 64 | 42 | 22 | **0.0024** |
| Stage III+IV | 136 | 58 | 78 |  |
| Lymph node metastasis |  |  |  |  |
| No | 73 | 45 | 28 | **0.0125** |
| Yes | 127 | 55 | 72 |  |
| Distant metastasis |  |  |  |  |
| No | 179 | 95 | 84 | **0.0112** |
| Yes | 21 | 5 | 16 |  |
| CEA (ng/ml) |  |  |  |  |
| <4.70 | 96 | 44 | 52 | 0.2575 |
| ≥4.70 | 104 | 56 | 48 |  |

NOTE: CEA carcinoembryonic antigen

P < 0.05 was considered significant. The bold type represents P values smaller than 0.05
