# Supplementary material for: UBE2J1 inhibits colorectal cancer progression by promoting ubiquitination and degradation of RPS3
Source: Oncogene. 2022 Dec 26;42(9):651–64. doi: 10.1038/s41388-022-02581-7 (PMC9957728; doi:10.1038/s41388-022-02581-7)
Supplement: Supplementary file 10 — Table S2 [file 41388_2022_2581_MOESM10_ESM.docx]

| **Table S2** | | |
| --- | --- | --- |
| **Antigens** | **Manufacturer** | **Application** |
| UBE2J1 | Santa Cruz Biotechnology: sc-377002 | 1:100 for WB; 1:50 for IHC; 2μg for IP |
| RPS3 | Abcam: ab140688 | 1:2000 for WB; 1:1000 for IHC; 4μg for IP |
| TRIM25 | Abcam: ab167154 | 1:2000 for WB; 1:40 for IP |
| P65 | Abcam: ab16502 | 1:1000 for WB |
| p-P65 | Abcam: ab194726 | 1:500 for WB; 1:50 for IHC |
| Ki-67 | Abcam: ab15580 | 1:500 for IHC |
| α-Tubulin | Proteintech: 66031-1-Ig | 1:20000 for WB |
| GAPDH | Abcam: ab9485 | 1:2500 for WB |
| Lamin B1 | Proteintech: 12987-1-AP | 1:2000 for WB |
| Flag | CST: #14793 | 1:1000 for WB; 1:50 for IP |
| Myc | CST: #2276 | 1:1000 for WB; 1:250 for IP |
| His | CST: #12698 | 1:1000 for WB; 1:50 for IP |
| HA | CST: #3724 | 1:1000 for WB |
